# Scalable synthesis of Cu clusters for remarkable selectivity control of intermediates in consecutive hydrogenation

**DOI:** 10.1038/s41467-023-36640-8

**Published:** 2023-02-27

**Authors:** Dawei Yao, Yue Wang, Ying Li, Antai Li, Ziheng Zhen, Jing Lv, Fanfei Sun, Ruoou Yang, Jun Luo, Zheng Jiang, Yong Wang, Xinbin Ma

**Affiliations:** 1 Key Laboratory for Green Chemical Technology of Ministry of Education, Collaborative Innovation Center of Chemical Science and Engineering, School of Chemical Engineering and Technology, Tianjin University, Tianjin, 300072 China; 2 Shanghai Synchrotron Radiation Facility, Shanghai Advanced Research Institute, Chinese Academy of Sciences, Shanghai, 201800 China; 3 State Key Laboratory of Materials Processing and Die & Mould Technology, School of Materials Science and Engineering, Huazhong University of Science and Technology, Wuhan, Hubei 430074 China; 4 Institute for New Energy Materials, School of Materials, Tianjin University of Technology, Tianjin, 300384 China; 5 Voiland School of Chemical Engineering and Bioengineering, Washington State University, Pullman, WA 99164 USA

**Keywords:** Synthesis and processing, Chemical engineering, Heterogeneous catalysis

## Abstract

Subnanometric Cu clusters that contain only a small number of atoms exhibit unique and, often, unexpected catalytic behaviors compared with Cu nanoparticles and single atoms. However, due to the high mobility of Cu species, scalable synthesis of stable Cu clusters is still a major challenge. Herein, we report a facile and practical approach for scalable synthesis of stable supported Cu cluster catalysts. This method involves the atomic diffusion of Cu from the supported Cu nanoparticles to CeO_2_ at a low temperature of 200 °C to form stable Cu clusters with tailored sizes. Strikingly, these Cu clusters exhibit high yield of intermediate product (95%) in consecutive hydrogenation reactions due to their balanced adsorption of the intermediate product and dissociation of H_2_. The scalable synthesis strategy reported here makes the stable Cu cluster catalysts one step closer to practical semi-hydrogenation applications.

## Abstract (web summary)

Scalable synthesis of stable Cu clusters for heterogeneous catalysis is still a major challenge. Here the authors report a low-temperature atomic diffusion approach for synthesis of stable Cu cluster catalysts, which exhibit high yield of intermediate product in consecutive hydrogenation reactions.

## Introduction

Supported Cu-based catalysts have been used in a variety of industrially important applications such as water-gas shift reaction^1^, methanol synthesis^2^, and hydrogenation of esters^3^ and CO_2_^4^. Understanding and control of catalytic activity of Cu species from single atoms (SAs) to nanoparticles (NPs) have thus generated a great deal of interest recently^5–7^. Of particular interest are subnanometric Cu clusters, containing only a small number of atoms, which can exhibit distinctive catalytic behavior because of their unique geometrical and electronical properties including the high density of active sites^8,9^, low-coordination evironment^10,11^, and appropriate electron-deficient surface^10,12–14^. Synthesis strategies for atomically tailoring Cu clusters by atomic layer desorption^10,11^, magnetron sputtering^15,16^, and electrospray ionization^17^ have emerged in recent years, but the low yields and high costs associated with these methods hinder their industrial applications. High mobility of Cu atoms makes it even more challenging to synthesize stable subnanometric Cu clusters in a facile and scalable way.

A promising strategy has recently been reported on synthesizing thermally stable single-atom catalysts via a facile thermal treatment, in which the supported metal particles^18,19^, bulk metals^20^, or metal oxides^21^ can atomically diffuse between supports and reconstruct to form stable SAs. However, high temperatures (e.g., 600–1000 °C) are required in this process to ensure the mobility of metal atoms and to enable atomic diffusion. A potential downside is that once the emitted metal atoms exceed the anchoring capacity under the high-temperature treatment conditions, they tend to form large metal NPs rather than SAs or subnanometric clusters^22–24^, which is difficult to tailor and maintain the monodispersity.

**Fig. 1:**
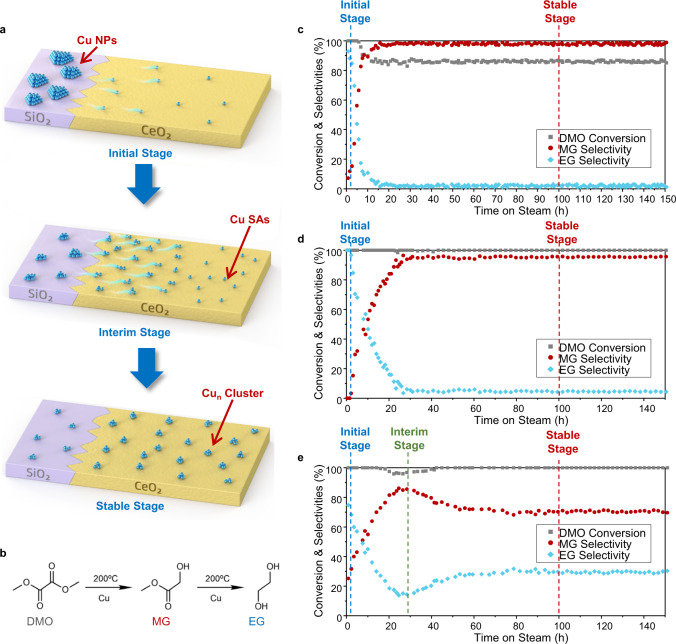
Cu atomic thermal diffusion process and its influence on catalytic performance in long-term DMO hydrogenation. **a** Scheme of Cu atomic thermal diffusion process in forming clusters. **b** Scheme of the consecutive hydrogenation of DMO to MG and EG. **c**–**e**, Catalytic performance of 3Cu (**c**), 5Cu (**d**), 7Cu (**e**) in long-term DMO hydrogenation. Reaction conditions: 200 °C, WLHSV_Cu_ = 6 h^−1^, H_2_/DMO = 80. The catalysts were made by physically mixing CeO_2_ and corresponding lamellar-structured copper silicates listed in Supplementary Table 1. Three different stages of catalysts (initial, interim, stable) during Cu low-temperature thermal diffusion are defined.

In this work, we report the synthesis of stable Cu subnanometric cluster catalysts via a low-temperature atomic diffusion process which are highly selective in consecutive hydrogenation to form desired intermediate products. By controlling the size of Cu NPs in the starting materials (e.g., Cu/SiO_2_), Cu atoms atomically diffuse from these NPs at low temperature (200 °C) and reconstruct to form stable clusters containing only a small number of atoms on CeO_2_ (Fig. 1a). The obtained supported Cu clusters exhibit superior semi-hydrogenation selectivity with excellent stability in various consecutive hydrogenation reactions, such as acetylene hydrogenation to ethylene and dimethyl oxalate (DMO) hydrogenation to methyl glycolate (MG, Supplementary Fig. 1). Furthermore, due to the facile synthesis of catalyst precursors, this low-temperature atomic diffusion strategy can be readily scaled-up and has been demonstrated in a large-batch synthesis of stable supported Cu catalysts with the control of Cu nuclearity from single atoms to clusters, showing promising prospects in industrial applications.

## Results and discussion

### The atomic diffusion of Cu species occurring at low temperature

To obtain stable Cu clusters via atomic thermal diffusion, it is important to select the temperature of thermal treatment to facilitate the detachment and diffusion of Cu atoms from supported Cu NPs with small and uniform size while preventing aggregation of Cu clusters. We previously reported the synthesis of lamellar-structured copper silicate by a facile ammonia-evaporation (AE) method^25^. Due to the confinement by lamellar structure, the obtained Cu NPs after reduction are uniformly dispersed with around 3 nm diameters (Supplementary Fig. 2). Here, catalysts were prepared by physically mixing the copper silicate precursors aforementioned with CeO_2_ which was obtained by calcination of cerium nitrate^18^, followed by reduction in pure H_2_ at 300 °C for 4 h and further treatment in pure H_2_ at 200 °C for 100 h. It was found from the element distribution (Supplementary Fig. 3) that, after low-temperature thermal treatment, most Cu species appeared and were highly dispersed on CeO_2_ rather than SiO_2_, evidencing the successful diffusion of Cu between these two supports and its reconstruction at low temperatures.

DMO hydrogenation has been found to be sensitive to the chemical states of Cu species^25,26^. This reaction contains two consecutive hydrogenations of the two symmetrical ester groups (DMO to MG, then MG to ethylene glycol (EG), as shown in Supplementary Fig. 1). These two reactions can happen under exactly the same conditions^27^, and usually the hydrogenation intermediate MG is not easily obtained over the copper-based catalyst because the thermodynamic constant of the second hydrogenation step is two orders of magnitude larger than that of the first hydrogenation step^28^, leading to both the DMO conversion and MG selectivity being sensitive to the changes of Cu sites. It is also noted that the DMO hydrogenation was carried out at the same temperature as the thermal treatment aforementioned (200 °C), thus we here used it as the probe reaction to reveal the evolution of Cu species during long-term reaction on a series of catalysts with increased Cu loadings (3, 5, and 7 wt% Cu shown in Fig. 1c–e, respectively). In the beginning of reaction, all catalysts exhibited high selectivity to EG (close to 100%) from the corresponding Cu NPs precursors^3^ (Supplementary Table 1). As the reaction proceeded, the selectivity of intermediate product MG gradually increased on all the catalysts, implying that the surface Cu is altered likely due to the atomic thermal diffusion of Cu under heat treatment. The performance of 3Cu and 5Cu catalysts stabilized after 10 h and 24 h, respectively, and remained unchanged in the subsequent >100 h test (Fig. 1c, d), indicating that the Cu diffusion process was completed and the stable Cu species were formed. Notably, evolution of catalytic performance over 7Cu catalyst (Fig. 1e) is different from those on 3Cu and 5Cu catalysts. After 29 h time-on-stream, EG selectivity slightly increased while MG selectivity slightly decreased, suggesting the Cu atomic diffusion in 7Cu catalyst continued, then stable catalyst performance was reached in the subsequent 50 to 150 h time-on-stream test. Based on the catalytic performance, the interim stage of Cu atomic diffusion could be probed. It is worth noting that the initial increase and then decrease of MG selectivity was even more obvious on the catalyst with further increased Cu loadings, e.g., 9Cu catalyst, on which the EG became the predominant product again after 100 h time-on-stream (Supplementary Fig. 4a). To investigate the influence of reactants on this Cu atomic diffusion process, we switched the inlet flow between the reactants (H_2_ + DMO) and the inert gas (N_2_) or pure H_2_ but kept the same heating program during the long-term reaction. As shown in Supplementary Fig.s 4b and c, similar catalytic performances were observed under different gas conditions, indicating that the assembly of Cu atoms is thermally driven. According to the catalytic performance in the long-term hydrogenation reaction, thermally treating stages can be defined and used for further characterization. The catalysts just after reduction (0 h) and after long-term thermal treatment (after 100 h) are denoted as reaching initial stage and stable stage, respectively, as marked in Fig. 1c–e. Furthermore, owing to the unique performance of 7Cu and 9Cu catalysts, an interim stage is defined at 29 h thermal treatment, as marked in Fig. 1e and Supplementary Fig. 4.

An additional experiment was performed to verify the diffusion of Cu between supports, which is illustrated in Supplementary Fig. 5. The Cu/SiO_2_ and CeO_2_ were shaped into pellets with different diameters of about 0.12 mm and 0.63 mm respectively, reduced separately and then physically mixed. After 100 h treatment in hydrogen under 200 °C, the CeO_2_ pellets were sifted out for evaluation. We found that the sieved CeO_2_ pellets are active in DMO hydrogenation, while the untreated CeO_2_ is inactive for DMO conversion. The Cu species on separated CeO_2_ pellets was detected by EDS-mapping (Supplementary Fig. 5b). These results clearly confirm that the atomic diffusion of Cu species from SiO_2_ to CeO_2_ occurs at a low temperature of 200 °C even in the absence of DMO reactant.

### Formation of Cu clusters during low-temperature atomic diffusion

**Fig. 2:**
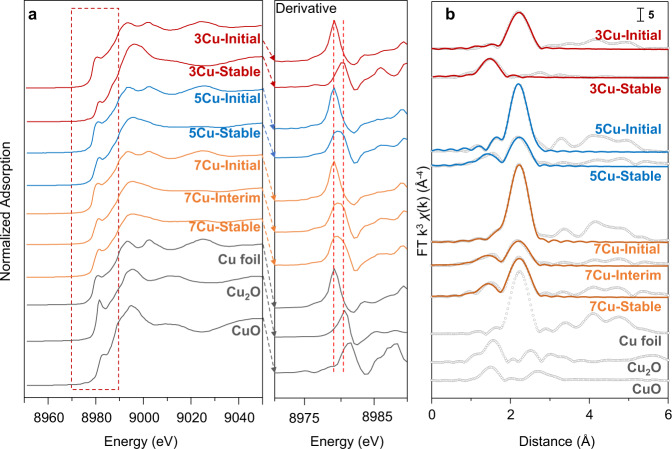
XAFS characterization of Cu species during atomic thermal diffusion process. **a** Cu K XANES spectra and the first derivatives of catalysts with different Cu loadings and stages, while the Cu K adsorption energies are listed in Table 1. **b** Cu R space EXAFS spectra of catalysts with different Cu loadings and treatment stages. The dots are original data and lines are fitted by FEFF code from the reported crystal structures^44^ (JCPDS card#: 4-836, 5-667, and 41-254 for Cu, Cu_2_O, and CuO respectively). The fitted structural data of Cu catalysts are shown in Table 1.

To investigate the atomic diffusion of Cu species under low-temperature thermal treatment at 200 °C, both X-ray absorption fine structure (XAFS) and atomic-resolution scanning transmission electron microscopy (STEM) characterizations were conducted. The X-ray absorption near edge structure (XANES) spectra and their corresponding first derivative data are shown in Fig. 2a. The corresponding Cu-K adsorption energies of all samples are listed in Table 1. The XANES and Cu-K adsorption energies of Cu-Initial catalysts are similar to the Cu foil (8979.0 eV), which indicates that all the Cu NPs before low-temperature thermal treatment are nearly at metallic state. After the low-temperature thermal treatment at 200 °C to the stable stage, Cu diffusion took place and the Cu-K adsorption energies of 3Cu, 5Cu, and 7Cu catalysts increased but are still lower than the Cu_2_O reference, indicating that part of the Cu species are present as Cu^δ+^ (0<δ ≤ 1) after anchoring on coordinately unsaturated CeO_2_ surface. It has been previously reported that the bottom layer of Cu NPs or clusters contains mainly Cu^δ+^ atoms that are chemically bonded with the Ce^3+^-O_v_ (oxygen vacancy), whereas the top layer of Cu NPs atoms has predominantly Cu^0^ atoms^29,30^. During the Cu atomic diffusion, the Cu species reconstructed to form single atoms or clusters on CeO_2_, resulting in increased Cu average valence.

**Table 1:** Cu-K adsorption energy and EXAFS fitting results of Cu catalysts in the different stages

Catalyst	Cu-K adsorption energy (eV)	Path	R (Å)^a^	CN^b^	σ^2^ (10^−3^ Å^2^)^c^	ΔE_0_ (eV)^d^	R-factor
3Cu-Initial	8979.0	Cu-Cu	2.53 ± 0.02	9.6 ± 0.9	9.6	3.95	0.007
		Cu-O	1.83 ± 0.02	1.0 ± 0.1	9.6		
3Cu-Stable	8980.5	Cu-Cu	−^e^	−	−	3.44	0.020
		Cu-O	1.85 ± 0.02	1.3 ± 0.1	7.6		
5Cu-Initial	8979.0	Cu-Cu	2.53 ± 0.02	8.9 ± 0.8	9.2	3.91	0.009
		Cu-O	−	−	−		
5Cu-Stable	8979.6	Cu-Cu	2.53 ± 0.02	2.6 ± 0.2	7.2	4.77	0.014
		Cu-O	1.85 ± 0.02	1.6 ± 0.2	7.2		
7Cu-Initial	8979.0	Cu-Cu	2.54 ± 0.02	8.5 ± 0.9	8.8	3.54	0.004
		Cu-O	−	−	−		
7Cu-Interim	8979.4	Cu-Cu	2.54 ± 0.02	3.5 ± 0.3	8.7	4.51	0.021
		Cu-O	1.85 ± 0.02	1.9 ± 0.2	8.3		
7Cu-Stable	8979.2	Cu-Cu	2.54 ± 0.02	4.8 ± 0.5	9.7	4.53	0.007
		Cu-O	1.85 ± 0.02	1.3 ± 0.1	5.2		
Cu^f^	8979.0	Cu-Cu	2.56	12			
		Cu-O					
Cu_2_O^f^	8980.8	Cu-Cu	3.70	8			
		Cu-O	1.85	4			
		Cu-Cu	2.91	4			
CuO^f^	8983.8	Cu-O	1.91	2			
		Cu-O	1.99	2			

^a^R is interatomic distance.

^b^CN is coordination number.

^c^σ^2^ is Debye-Waller factor, a measure of thermal and static disorder in absorber scatter distances.

^d^ΔE_0_ is edge energy shift.

^e^No reliable fitted values can be obtained.

^f^EXAFS data (R and CN) were calculated by FEFF code from the reported crystal structures^44^ (JCPDS card#: 4-836, 5-667, and 41-254 for Cu, Cu_2_O, and CuO, respectively).

We further obtained the structural and coordination information of Cu species according to the extended X-ray absorption fine structure (EXAFS) R space spectra (Fig. 2b) and the corresponding fitting results of coordination numbers (CN, listed in Table 1). For all Cu-Initial catalysts, the main peaks of Cu-Cu at 2.24 Å with coordination number of 8.5–9.6 were observed, which is consistent with the existence of 2–3 nm Cu NPs in the initial stage^31^. After low-temperature thermal treatment, the structure of Cu species apparently changed. For 3Cu-Stable, the Cu-Cu peak disappeared while the Cu-O peak at 1.50 Å appeared, indicating that the Cu NPs restructured to form Cu SAs during the thermal diffusion. The formation of Cu SAs proved that atomic diffusion rather than particle migration occurred during the low-temperature thermal treatment, which might be benefited from the well-uniformed size distribution of Cu NPs in the initial precursors synthesized using the AE method^25^.

**Fig. 3:**
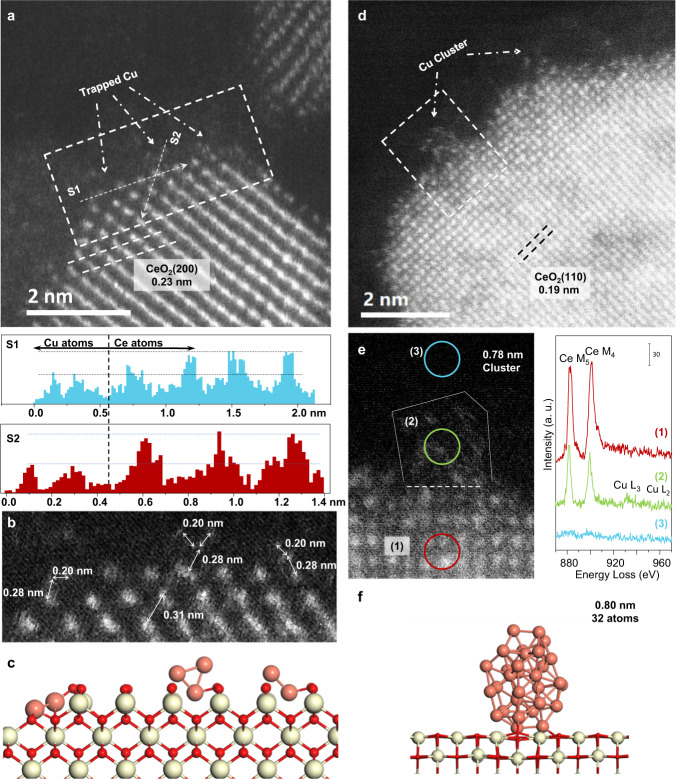
Structural information of Cu clusters in 5Cu-Stable and 7Cu-Stable. **a** HAADF-STEM image of 5Cu-Stable and intensity line scans from two directions (S1 and S2) of the top atomic layers of CeO_2_. The top three layers of CeO_2_ edge (8 × 3 atoms) surface was magnified as shown in (**b**). **b** Distance between atoms. The distance between the darker atoms and the brighter atoms are 0.20 nm and 0.31 nm respectively, corresponding to the interatomic distance of Cu-Cu and Ce-Ce^1^. **c** Corresponding theoretical structure of (**b**), optimized by DFT. **d** HAADF-STEM image of 7Cu-Stable, the interface between Cu cluster and CeO_2_ can be distinguished according to the line scans as shown in Supplementary Fig. 7b. **e** STEM-EELS of different areas of 7Cu-Stable. **f** Corresponding optimized theoretical structure by DFT calculation.

**Fig. 4:**
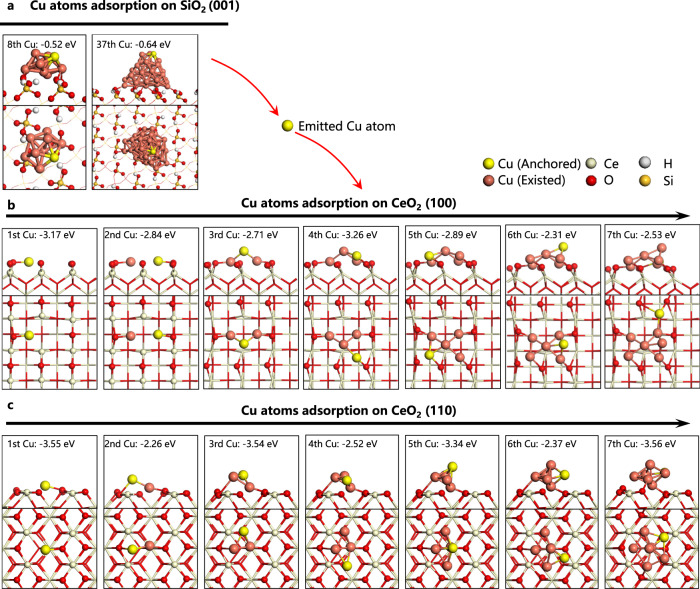
The formation process of Cu clusters during ordered atomic diffusion investigated by DFT study. **a** Cu adsorption energies on Cu clusters and NPs on SiO_2_. **b**, **c** The most stable adsorption positions of Cu atoms on CeO_2_ (100) (**b**) and CeO_2_ (110) (**c**) surface and their relative adsorption energies. All of the potential positions for Cu atom absorbed on CeO_2_ (100) and CeO_2_ (110) surface and corresponding adsorption energies are shown in Supplementary Figs. 12–15.

By fitting the EXAFS of catalysts with different Cu loadings and low-temperature thermal treatment stages, the Cu atomic diffusion process could be further elucidated. According to the coordination number of 2.62 and 4.84 in 5Cu-Stable and 7Cu-Stable, respectively, it could be deduced that the Cu clusters of 2–3 atoms formed in 5Cu-Stable while those in 7Cu-Stable contain about 30 atoms^32^. The arrangements of copper atoms within clusters in 5Cu-Stable and 7Cu-Stable were further confirmed by atomic-resolution high-angle annular dark-field (HAADF) STEM combined with electron energy loss spectroscopy (EELS). Unlike the noble metal atoms, Cu atoms are difficult to be imaged on the CeO_2_ background, thus the edge of CeO_2_ was focused to detect the metal atoms with low atomic number^1,33^. We here conducted STEM and EELS for several CeO_2_ edges to determine the morphology and uniformity of Cu species, as shown in Fig. 3 and Supplementary Figs. 6–8. The intensity line scanning of the edge of 5Cu-Stable (Fig. 4a and Supplementary Fig. 6) and 7Cu-Stable (Supplementary Fig. 7b) shows the location of Cu atoms and Ce atoms, demonstrating the Cu clusters are on the CeO_2_ surface. The distance of the adjoining copper atoms on the top layer was 0.20 nm, which is equal to the interplanar spacing of 0.208 nm in Cu(111). The distance between the Ce atoms of the top layer and the Cu atoms was 0.28 nm, demonstrating that Ce and Cu atoms in the interface are connected via interfacial O atoms which are invisible in the HAADF-STEM image^1^. The Cu L_2,3_ and Ce M_4,5_ edges of line-scanning EELS in Fig. 3e and Supplementary Fig. 8 further affirmed the existence of Cu clusters on CeO_2_ surface. It could be seen that the trapped Cu species present mainly as clusters of 2–3 atoms on 5Cu-Stable catalyst, in accordance with the EXAFS fitting results in Table 1. Furthermore, large-scale STEM-EDS images of 5Cu-Stable catalyst and the corresponding fast Fourier transform image are shown in Supplementary Fig. 10. The Cu atoms are located on CeO_2_, while there is no lattice fringe of any Cu planes in the same region, confirming the high dispersion of Cu species. The geometric configuration of clusters on 5Cu-Stable (2–3 atoms) and 7Cu-Stable (0.78 nm) are further built by density functional theory (DFT) respectively, as shown in Figs. 3c and 3f. The Cu atoms at the Cu-CeO_2_ interface are calculated of positive charge^1^, which is also demonstrated by XANES (Fig. 2a), which confirmed the appearance of Cu^δ+^ (0 < δ ≤ 1) after anchoring on coordinately unsaturated CeO_2_ surface. The optimized 0.80 nm cluster is formed by DFT (Fig. 3f), which is consistent with the EXAFS fitting result of 7Cu-Stable (Table 1). Notably, compared to the CeO_2_ supported Cu clusters prepared by deposition-precipitation method^1^, these Cu clusters generated via the atomic diffusion are more like hemispheres rather than monolayers or bilayers. To further clarify that the Cu diffusion is thermally driven, we treated 5Cu and 7Cu catalysts with the same heating program but in the absence of reactants, i.e., these two reduced catalysts were treated under N_2_ at 200 °C for 100 h. The HAADF-STEM images of these two samples (shown in Supplementary Fig. 9) demonstrate that Cu atoms can also diffuse to CeO_2_ and form Cu clusters after thermal treatment in the absence of reactants.

It can be deduced the Cu SAs are generated in the 3Cu-Stable by Cu atomic diffusion and anchoring on the defects of CeO_2_ surface during thermal treatment^18,34^. However, when excess Cu are available in the precursor, we found that the atomic thermal diffusion would continue to form Cu clusters, such as what occurred in the 5Cu-Stable and 7Cu-Stable catalysts. The Cu-Cu coordination number of 7Cu sample first decreased before the interim stage but then increased in the following 70 h treatment (Table 1). It also could be seen from the Cu-K adsorption energy (Table 1) that the average valence of Cu species increased first but then decreased. This changing pattern is more obvious when we increased the initial Cu content to 9 wt%, where the size of Cu species decreased at first, and then increased, eventually forming Cu NPs after the 100 h low-temperature thermal treatment, as shown in Supplementary Fig. 11 and Supplementary Table 2. Accordingly, the formation processes of clusters and NPs during low-temperature atomic diffusion could be deduced. When all the available sites for trapping Cu atoms are occupied, excess Cu atoms would continue to be emitted, diffuse, and deposit around the trapped single-atom sites, forming clusters or NPs.

To further confirm the Cu atomic diffusion mechanism, DFT calculation was conducted to investigate the detachment of Cu atoms from cluster supported on SiO_2_ and the anchor of these detached Cu atoms on CeO_2_(100) and CeO_2_(110). As shown in Fig. 4 and Supplementary Figs. 12–15, the adsorption energies of Cu atoms anchored on CeO_2_ are −2.26 eV~−3.56 eV, which are much lower than those adsorption energies on SiO_2_ (−0.52~−0.64 eV). This result indicates Cu atoms are more stable on CeO_2_ surface than SiO_2_ surface, and confirms that the detached Cu atoms from SiO_2_ prefers to anchor on the CeO_2_ surfaces. According to the potential anchoring positions of Cu atoms (Supplementary Figs. 12–15), the Cu atoms tend to first being stabilize on oxygen vacancy of CeO_2_ to form single Cu sites, followed by additional Cu atoms migrating on the surface and anchoring at the existing Cu sites, leading to the growth of the Cu clusters on both CeO_2_(100) and CeO_2_(110) facet. We could also find that the Cu_1_ sites and Cu_3~4_ clusters are more stable than other sites during the anchoring process, which is consistent with our finding from EXAFS and STEM that single Cu site and Cu_3_ cluster are formed under the low Cu loadings.

Based on these experimental and theoretical results, it could be concluded that the stable Cu clusters are obtained via a two-step atomic diffusion process at low temperature, as illustrated in Fig. 1a. The first step, which happens in the initial several hours, involves Cu atoms emitting from NPs precursors and then being trapped by the defects on CeO_2_ to form stable Cu SAs. During this step, the average crystalline size of Cu decreases while the surface proportion of atomically dispersed Cu^δ+^ increases. After all the available sites on CeO_2_ are occupied, emitted excess Cu atoms would anchor around the single atoms, gradually forming stable Cu clusters.

### Subnanometric Cu clusters for efficient semi-hydrogenation

During the long-term DMO hydrogenation, the selectivity of semi-hydrogenation product and final product changed significantly as the Cu catalysts evolved through Cu atomic diffusion. Based on the results discussed above, it could be deduced that the size of Cu domains, especially clusters, could significantly influence their catalytic behavior in consecutive hydrogenations. To further confirm this hypothesis, we prepared a series of stable Cu catalysts with different domain sizes via the low-temperature atomic diffusion method by modulating the Cu loadings. As confirmed by the XRD, EXAFS, and STEM-EDS characterization (Supplementary Figs. 16–18 and Supplementary Table 3), Cu NPs of 4.1 nm and 7.0 nm were synthesized on 9Cu-Stable and 11Cu-Stable catalysts, respectively, as well as the Cu SAs (3Cu-Stable) and Cu clusters (i.e., Cu_3_ clusters (5Cu-Stable) and Cu_30_ clusters (7Cu-stable)). It is worth noting that these catalysts can be readily scale-up prepared (at least 1 kg per batch, as demonstrated in Supplementary Fig. 19), showing promising prospects in industrial application.

**Fig. 5:**
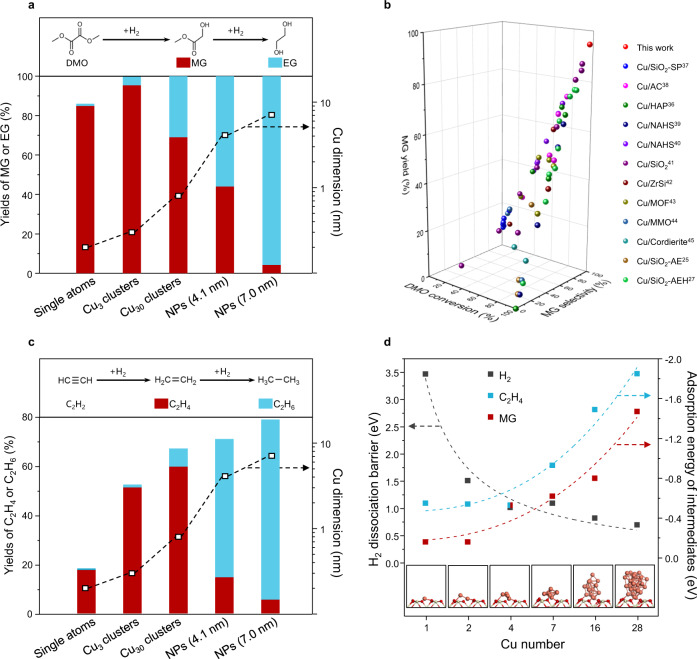
Regulating the selectivity of hydrogenation by controlling the dimension of Cu species at atomic scale. **a** Catalytic performance of Cu species with different domain sizes for DMO hydrogenation. Reaction conditions: 200 °C, 2.5 MPa, H_2_/DMO = 80, flow rate of 6 g_DMO_/(g_Cu_·h) normalized based on amount of exposed Cu weight. **b** Summary of DMO conversion, MG selectivity, and MG yield obtained over Cu-based catalysts in literatures, including Cu/SiO_2_-SP^36^, Cu/AC^51^, Cu/HAP^35^, Cu/NAHS^52,53^, Cu/SiO_2_^39^, Cu/ZrSi^54^, Cu/MOF^55^, Cu/MMO^56^, Cu/Cordierite^57^, Cu/SiO_2_-AE^25^, and Cu/SiO_2_-AEH^27^. Detailed reaction information is shown in Supplementary Table 4. **c** Catalytic performance of Cu species with different dimensions for C_2_H_2_ hydrogenation. Reaction condition: 120 °C, 0.1 MPa, 1% acetylene, 10%H_2_ balanced in Ar. 0.956 g_C2H2_/(g_Cu_·h) normalized based on amount of exposed Cu weight. By-products: C_3-4_ oligomers. **d** H_2_ diss °Ciation barrier, MG adsorption energy, and C_2_H_4_ adsorption energy on Cu species with different sizes. Detailed DFT results are shown in Supplementary Figs. 21–24.

Two representative consecutive hydrogenation reactions, DMO hydrogenation and acetylene hydrogenation, were carried out to understand the catalytic performance over Cu SAs, Cu clusters, and Cu NPs (Fig. 5). The flow rate of reactant is set according to Cu surface amount to fix the ratio between exposed Cu sites and the amount of reactant per unit time. The catalytic performance of DMO hydrogenation is shown in Fig. 5a. For single-atom Cu catalyst, the semi-hydrogenation product MG is predominant with 84% yield. As the size of Cu species increased from single atom to Cu_3_ cluster, the MG yield increased from 84% to 95% (95% MG selectivity and 100% DMO conversion). With further increasing the Cu dimension, the DMO conversion reached 100% but the MG selectivity gradually decreased because of the further hydrogenation of MG to EG. The EG selectivity consequently increased and reached 95% when the size of Cu NPs reached about 7 nm. Thus, by simply tailoring the domain size of Cu species, the yield of semi-hydrogenation product MG could be improved to 95% in DMO hydrogenation. According to previous reported works on Cu-based catalysts, the high MG selectivity could only be achieved by increasing space velocity of DMO^27^, decreasing reaction temperature^35^ or reducing the amount of exposed Cu species^36^. However, these approches unavoidably led to drastically decreased DMO conversion, and consequently low MG yield. The catalytic performance data of all previous literature reports are summarized in Fig. 5b, showing that all MG yields are lower than 80%. Notably, the catalytic performance of Cu_3_ cluster in this work shows a distinctively high MG selectivity of 95% without sacrificing the DMO conversion (100%), resulting in an unprecedented MG yield of 95%. The clusters also displayed remarkable catalytic performance in semi-hydrogenation of acetylene to ethylene shown in Fig. 5c. It could be seen that the conversion of acetylene over Cu SAs was much lower than that over clusters, and the selectivity of ethylene decreased sharply when the Cu species increased from clusters to NPs. Based on the catalytic performance in these two consecutive hydrogenation reactions, it is apparent that the Cu clusters exhibit high selectivity to intermediate products even at high conversions, resulting in significantly improved yield of semi-hydrogenation products.

Selective hydrogenation remains a general challenge in chemical synthesis, which is usually plagued by the rapid over-hydrogenation^37,38^. To better understand the superior performance of Cu clusters in semi-hydrogenation, we investigated the adsorption and activation behavior of the intermediate products (MG and ethylene) and H_2_ on Cu species of different dimensions (Fig. 5d). As shown in the MG temperature programed desorption combined with mass spectrometry (Supplementary Fig. 20), the MG desorption peak shifted from 150 °C to 171 °C with the size of Cu species increased from single atom to 7 nm, indicating that the adsorption of MG is stronger on larger Cu species. We also investigated the MG desorption and the first step of MG hydrogenation (MG to MGH*)^39^ via DFT calculation, as shown in Supplementary Figs. 21 and 22. With the increased Cu atoms number of the active sites, the MG adsorption energies gradually decrease from −0.16 eV (Cu_1_) to −1.48 eV (Cu_28_), indicating that MG could be more easily desorbed from smaller Cu sites. The activation barrier of MG to MGH* (first MG hydrogenation step) only slightly changed when varying the Cu atoms number, indicating that MG hydrogenation ability is not the primary factor for influencing the MG selectivity. It is therefore deduced that the Cu sites with only a few atoms tend to release MG instead of further hydrogenate them to EG, resulting in a higher MG yield. The desorption behavior of ethylene on Cu species with different dimensions is similar to MG desorption, as shown in Supplementary Fig. 23. The H_2_ dissociation barrier on Cu species was calculated as well (Supplementary Fig. 24). With the increased Cu size, the H_2_ dissociation barrier gradually decreased, indicating more readily dissociation of H_2_ molecules on larger Cu species^40–42^. From both experimental and computational results, it could be inferred that the low H_2_ dissociation ability of Cu SAs is the main reason of the low conversion in hydrogenation reactions. On the other hand, the strong adsorption of intermediate products (MG and ethylene) on Cu NPs leads to the over-hydrogenation to the final products (EG and ethane), which is the main reason of low selectivity to intermediate products. Cu clusters, with high H_2_ dissociation ability and relative low adsorption ability towards intermediate products, are shown to be effective for semi-hydrogenation reactions.

In this work, we proposed a simple and practical strategy to synthesize stable Cu clusters containing only a small number of atoms, in which the Cu atoms could detach from supported nanoparticles, atomically diffuse and restructure on CeO_2_ surface under low temperature (200 °C). The Cu clusters exhibited remarkable catalytic performance in selective hydrogenations to producing intermediate products, such as DMO hydrogenation to MG or acetylene hydrogenation to ethylene. In particular, the Cu_3_ clusters achieved unprecedented MG yields as high as 95%. Combined experimental and computational studies show that the stable Cu clusters exhibit high H_2_ dissociation ability and low adsorption ability towards intermediate products, resulting in the efficient semi-hydrogenation performance. Furthermore, this low-temperature atomic diffusion strategy is readily scaled-up and we have demonstrated in producing kilogram-batch catalysts with Cu size regulated ranging from single atoms to clusters, which shows great potential in semi-hydrogenation at industrial level.

## Methods

### Synthesis of the lamellar-structured copper silicate

Lamellar-structured copper silicate was prepared by ammonia evaporation (AE) method. Cu(NO)_2_•3H_2_O (99.99%; Sigma-Aldrich) and ammonia water (25 wt%; J&K Co.) were first dissolved into the deionized water and stirred for 10 min to form cuprammonia solution. The silica sol was dropwise added into the solution and stirred for 4 h. Then this mixture was heated in an 80 °C water bath to allow the evaporation of ammonia and consequent precipitation. The evaporation process was terminated when the pH value decreased to 7. The obtained product was filtered, washed, dried under vacuum at 80 °C, and calcined in air at 450 °C for 4 h to obtain the light blue powder sample.

### Synthesis of CeO_2_

The Ce(NO_3_)_3_•6H_2_O (99.999%; Sigma-Aldrich) was heated in air at 350 ˚C for 2 h, producing the yellow powder sample.

### Physically mixed samples

The lamellar-structured copper silicate and CeO_2_ were mixed at a weight ratio of 1:3.6 and ground for 15 min, then pressed and crushed into granules of 40–60 meshes.

### Catalytic activity test

Catalytic activity tests were carried out in a straight stainless-steel fixed-bed reactor with an internal diameter of 8 mm. The reactor was placed inside a vertical furnace with three temperature controllers. 0.2–1 g catalyst (40–60 meshes) was loaded into the reactor between two layers of quartz wools.

To investigate the catalyst evolution process during long-term DMO hydrogenation, the as-prepared catalysts were first reduced by hydrogen at 300 °C for 4 h then decreased to 200 °C, and then the feed with a H_2_/DMO molar ratio of 80 was injected into the reactor. The DMO flow rate was set according to the total Cu weight, which is 6 g_DMO_ g_Cu_^−1^ h^−1^. Three stages are defined for the catalysts according to the reaction time-on-stream at 200 °C: initial stage (after reduction; 0 h treatment at 200 °C), interim stage (29 h treatment at 200 °C), and stable stage (100 h treatment at 200 °C).

To investigate the influence of Cu domain size on the consecutive hydrogenations, the as-prepared catalysts with different Cu loadings (x%) were first reduced by hydrogen at 300 °C for 4 h, then treated at 200 °C in hydrogen for 100 h to reach the stable stage, denoted as xCu-Stable. For DMO hydrogenation, the DMO flow rate was set according to the surface amount of Cu, which is 6 g_DMO_/(g_Cu_·h), and the H_2_/DMO molar ratio was kept at 80. The DMO hydrogenation was conducted at 200 °C and 2.5 MPa. For C_2_H_2_ hydrogenation, the C_2_H_2_ flow rate was also set according to the surface amount of Cu, which is 0.956 g_C2H2_/(g_Cu_·h), and the feed gas contains a mixture of 1 vol% C_2_H_2_ and 10 vol% H_2_ balanced with Ar. The C_2_H_2_ hydrogenation was conducted at 120 °C and 0.1 MPa.

The products in DMO hydrogenation were condensed and analyzed using an Agilent Micro GC 6820 chromatography with flame ionization detector (FID) and a HP-INNOWAX capillary column (Hewlett-Packard, 30 m × 0.32 mm × 0.50 μm). The vent gases in C_2_H_2_ hydrogenation were analyzed by a Shimadzu GC 2014C online gas chromatography with two thermal conductivity detectors (TCD) and one FID, employing an Al_2_O_3_ capillary column (25 m × 0.32 mm) and two packed columns (Molecular sieve-13X, 3.0 m × 3.2 mm and Porapak-N, 1.0 m × 3.2 mm).

### Characterization

The copper content of samples was analyzed by using an Inductively Coupled Plasma Optical Emission Spectrometer (ICP-OES, Varian Vista-MPX), and was determined by the Cu characteristic peak at 324.754 nm. Transmission electron microscopy (TEM) images and corresponding EDS of samples were obtained by a JEM-2100f system electron microscope at an electron acceleration voltage of 200 kV. Atomic resolution high-angle annular dark-field (HAADF) STEM images and corresponding EDS of samples were obtained by Titan Cubed Themis G2300 in Center for Electron Microscopy and Tianjin Key Lab of Advanced Functional Porous Materials, Tianjin University of Technology.

X-ray diffraction (XRD) was conducted by a Rigaku C/max-2500 diffractometer, employing the graphite-filtered Cu Kα radiation (λ = 1.5406 Å) at room temperature. The samples were scanned from 10° to 90° with a rate of 8°/min or from 30° to 50° with a rate of 2°/min.

X-ray adsorption fine structure (XAFS) profiles at E_0_ = 8979 eV (Cu K-edge) were obtained at BL14W1 beamline of Shanghai Synchrotron Radiation Facility (SSRF)^43^ operated at 3.5 GeV under top-up mode with a constant current of 220 mA. Before each measurement, the samples were placed in a quartz tube oven with valves at both ends and were reduced at 300 °C in pure hydrogen followed by further thermal treatment at 200 °C in hydrogen for different time. After these treatments, the quartz tube was directly sealed by the valves and transferred into the glovebox. Then the sample was taken out from the quartz tube and placed into a hollow Teflon chamber which was completely sealed by several layers of Kapton foil. This sealed chamber was directly placed in beamline for characterization. The obtained XAFS data was processed by standard procedures via ATHENA module in IFEFFIT software^44^. The EXAFS data was obtained by subtracting the background of post-edge and normalized by the edge jump step. The R-space data was obtained by Fourier transformed by the k-space data. The coordination information can be obtained by the least-squares curve fitting in the R-space using the ARTEMIS module in IFEFFIT software^44^.

The surface amount of Cu species in xCu-Stable catalysts was calculated according to Eq. (1):

1 $${{{{{\rm{Surface}}}}}}\,{{{{{\rm{amount}}}}}}\,{{{{{\rm{of}}}}}}\,{{{{{\rm{Cu}}}}}}\,{{{{{\rm{per}}}}}}\,{{{{{\rm{gram}}}}}}\,{{{{{\rm{catalyst}}}}}}={{{{{\rm{Cu}}}}}}\,{{{{{\rm{dispersion}}}}}}\\ \times {{{{{\rm{Cu}}}}}}\,{{{{{\rm{loading}}}}}}\,({{{{{\rm{g}}}}}}/{{{{{{\rm{g}}}}}}}_{{{{{{\rm{cat}}}}}}})$$

where Cu dispersion is calculated from Eq. (2)^45^:

2 $${{{{{\rm{Cu}}}}}}\,{{{{{\rm{dispersion}}}}}}\,(\%)=(6/{{{{{\rm{d}}}}}})\times ({{{{{{\rm{V}}}}}}}_{{{{{{\rm{A}}}}}}}/{{{{{{\rm{C}}}}}}}_{{{{{{\rm{A}}}}}}})$$

where V_A_ is Cu atomic volume in the crystal lattice (0.0119 nm^3^) and C_A_ is cross-sectional area of Cu atom (0.0680 nm)^45^.

MG temperature desorption profiles were obtained by a Micromeritics Autochem II 2920 apparatus. The catalysts with normalized weight according to Cu loading were first reduced at 300 °C for 4 h and further kept at 200 °C in hydrogen for different time. Then, the temperature was decreased to 100 °C in helium. MG was bubbled under 80 °C and carried by helium into the sample tube for 60 min. After MG adsorption the sample tube was purged by He under 100 °C for 30 min. Finally, the temperature was gradually increased to 400 °C at a rate of 5 °C/min in helium. The vent gas was analyzed by mass spectrometry (MS, Hiden HPR-20 EGA).

### Computational methods

Density functional theory (DFT) calculations were performed using the CASTEP package of Materials Studio with a planewave energy cutoff of 400 eV. The electron exchange and correlation were described by the generalized gradient approximations (GGA)^46^ with the Perdew−Burke−Ernzerhof (PBE) functional^46^. The Hubbard U term was used to properly localize the state of Ce 4f^47^. The effective U (U_eff_) parameter is defined as the difference between Coulomb U and exchange J. The U_eff_ of Ce was set to 4.5 eV for all the calculations^48,49^.

For the Cu/CeO_2_ catalyst, a seven-layer CeO_2_ (110) – (2 × 2) (11.14 Å × 7.55 Å) and a seven-layer CeO_2_ (100) – (3 × 3) (11.62 Å × 11.62 Å) slab with vacuum region of 15 Å between slabs were employed to simulate the CeO_2_ surfaces with different facet. The top two layers were allowed to relax while the bottom five were fixed to their bulk positions. The k-point mesh of 2 × 3 × 1 was used to generate the Brillouin zone.

For the Cu/SiO_2_ catalyst, we chose the (001) surface of α-quartz to simplify the surface structure of amorphous SiO_2_ in experiment because the SiO_2_ (001) surface has the lowest energy in low-index surface^50^. An eleven-layer SiO_2_ (001) – (4 × 4) (19.92 Å × 19.92 Å) slab with a vacuum space of 15 Å was employed to simulate the SiO_2_ surface. The top three layers were allowed to relax while the bottom eight were fixed to their bulk positions. Besides, half of the surface terminal O atoms were saturated by H atoms. Due to the large size of surface cells and large size of Cu_n_ clusters, the calculations were performed at a Γ-point grid.

The adsorption energy (Eads) of Cu was determined based on the energy difference by adding one more Cu atom to the existing Cu domain on CeO2 surface, as shown in the following Eq. (3),

3 $${{{{{{\rm{E}}}}}}}_{{{{{{\rm{ads}}}}}}}={{{{{{\rm{E}}}}}}}_{({{{{{\rm{slab}}}}}}+{{{{{\rm{nCu}}}}}})}-{{{{{{\rm{E}}}}}}}_{[{{{{{\rm{slab}}}}}}+({{{{{\rm{n}}}}}}-1){{{{{\rm{Cu}}}}}}]}-{{{{{{\rm{E}}}}}}}_{({{{{{\rm{Cu}}}}}})}$$

where E_(slab+nCu)_ and E_[slab+(n−1)Cu]_ represent the total energies of the slab with nCu cluster and (n–1)Cu cluster, respectively. A more negative E_ads_ indicates a stronger adsorption.

For ethylene adsorption, MG adsorption, MG hydrogenation, and H_2_ dissociation calculation, we chose a seven-layer CeO_2_ (110) – (2 × 4) slab (11.14 Å × 14.96 Å) with a 25 Å vacuum to simulate the surface of CeO_2_ support. The top two layers were allowed to relax while the bottom five were fixed to their bulk positions. The diameter of largest Cu cluster (Cu_28_) in xy direction is 6.48 Å. Due to the large size of surface cells and large size of Cu cluster, the calculations were performed at a k-point of Γ-point. the atomic structures were relaxed until the forces on all unconstrained atoms were less than 0.05 eV/Å, and the convergence criterion for energy was 2 × 10^−5^ eV. The adsorption energies (E_ads_) of MG and ethylene were defined as Eqs. (4) and (5) as follows,

4 $${{{{{{\rm{E}}}}}}}_{{{{{{\rm{ads}}}}}}}={{{{{{\rm{E}}}}}}}_{({{{{{\rm{surface}}}}}}+{{{{{\rm{MG}}}}}})}-{{{{{{\rm{E}}}}}}}_{({{{{{\rm{surface}}}}}})}-{{{{{{\rm{E}}}}}}}_{({{{{{\rm{MG}}}}}})}$$

5 $${{{{{{\rm{E}}}}}}}_{{{{{{\rm{ads}}}}}}}={{{{{{\rm{E}}}}}}}_{({{{{{\rm{surface}}}}}}+{{{{{\rm{C2H4}}}}}})}-{{{{{{\rm{E}}}}}}}_{({{{{{\rm{surface}}}}}})}-{{{{{{\rm{E}}}}}}}_{({{{{{\rm{C2H4}}}}}})}$$

where the E_(surface)_, E_(surface+MG)_, and E_(surface+C2H4)_ represent the total energy of Cu/CeO_2_(110) surface, Cu/CeO_2_(110) surface with absorbed MG, and Cu/CeO_2_(110) surface with adsorbed ethylene, respectively. A lower E_ads_ demonstrates the product is more stable on Cu/CeO_2_ surface. The activation barrier of MG to MGH* and H_2_ decomposition is calculated by the energy of transition state (E_(TS)_) minus the energy of Cu/CeO_2_(110) with adsorbed MG + H or adsorbed H_2_, as shown in the Eqs. (6) and (7),

6 $${{{{{{\rm{E}}}}}}}_{{{{{{\rm{act}}}}}}}={{{{{{\rm{E}}}}}}}_{({{{{{\rm{TS}}}}}})}-{{{{{{\rm{E}}}}}}}_{({{{{{\rm{MG}}}}}}+{{{{{\rm{H}}}}}})}$$

7 $${{{{{{\rm{E}}}}}}}_{{{{{{\rm{act}}}}}}}={{{{{{\rm{E}}}}}}}_{({{{{{\rm{TS}}}}}})}-{{{{{{\rm{E}}}}}}}_{({{{{{\rm{surface}}}}}}+{{{{{\rm{H2}}}}}})}$$

## Supplementary information

Supplementary Information

## Data Availability

The data generated in this study are provided in the Supplementary Information. More detailed data that support the findings of this study are available from the corresponding author upon reasonable request.

## References

[1] Chen A (2019). Structure of the catalytically active copper–ceria interfacial perimeter. Nat. Catal..

[2] Behrens M (2012). The active site of methanol synthesis over Cu/ZnO/Al_2_O_3_ industrial catalysts. Science.

[3] Yue H (2013). A copper-phyllosilicate core-sheath nanoreactor for carbon-oxygen hydrogenolysis reactions. Nat. Commun..

[4] Graciani J (2014). Highly active copper-ceria and copper-ceria-titania catalysts for methanol synthesis from CO_2_. Science.

[5] Li F (2018). Boosting oxygen reduction catalysis with abundant copper single atom active sites. Energy Environ. Sci..

[6] Gawande M (2016). Cu and Cu-based nanoparticles: Synthesis and applications in review catalysis. Chem. Rev..

[7] Wang Y (2018). Single-atomic Cu with multiple oxygen vacancies on ceria for electrocatalytic CO_2_ reduction to CH_4_. ACS Catal..

[8] Yang X (2013). Single-atom catalysts: A new frontier in heterogeneous catalysis. Acc. Chem. Res..

[9] Tyo E, Vajda S (2015). Catalysis by clusters with precise numbers of atoms. Nat. Nanotechnol..

[10] Liu C (2015). Carbon dioxide conversion to methanol over size-selected Cu_4_ clusters at low pressures. J. Am. Chem. Soc..

[11] Yang B (2017). Copper cluster size effect in methanol synthesis from CO_2_. J. Phys. Chem. C..

[12] Jiao J (2019). Copper atom-pair catalyst anchored on alloy nanowires for selective and efficient electrochemical reduction of CO_2_. Nat. Chem..

[13] Wang W (2017). Crystal plane effect of ceria on supported copper oxide cluster catalyst for CO oxidation: Importance of metal–support interaction. ACS Catal..

[14] Wang W (2015). Highly dispersed copper oxide clusters as active species in copper-ceria catalyst for preferential oxidation of carbon monoxide. ACS Catal..

[15] Schmidt M, Kusche R, von Issendorff B, Haberland H (1998). Irregular variations in the melting point of size-selected atomic clusters. Nature.

[16] Haberland H (1994). Filling of micron‐sized contact holes with copper by energetic cluster impact. J. Vac. Sci. Tech. A.

[17] Schröder D, Weiske T, Schwarz H (2002). Dissociation behavior of Cu(urea)^+^ complexes generated by electrospray ionization. Inter. J. Mass Spectrom..

[18] Jones J (2016). Thermally stable single-atom platinum-on-ceria catalysts via atom trapping. Science.

[19] Nie L (2017). Activation of surface lattice oxygen in single-atom Pt/CeO_2_ for low-temperature CO oxidation. Science.

[20] Qu Y (2018). Direct transformation of bulk copper into copper single sites via emitting and trapping of atoms. Nat. Catal..

[21] Yang Z (2019). Directly transforming copper (I) oxide bulk into isolated single-atom copper sites catalyst through gas-transport approach. Nat. Commun..

[22] Kunwar D (2019). Stabilizing high metal loadings of thermally stable platinum single atoms on an industrial catalyst support. ACS Catal..

[23] Hansen T, DeLaRiva A, Challa S, Datye A (2013). Sintering of catalytic nanoparticles: particle migration or ostwald ripening?. Acc. Chem. Res..

[24] Prieto G, Zecevic J, Friedrich H, de Jong K, de Jongh P (2013). Towards stable catalysts by controlling collective properties of supported metal nanoparticles. Nat. Mater..

[25] Gong J (2012). Synthesis of ethanol via syngas on Cu/SiO_2_ catalysts with balanced Cu^0^-Cu^+^ sites. J. Am. Chem. Soc..

[26] Zheng J (2022). Ambient-pressure synthesis of ethylene glycol catalyzed by C_60_-buffered Cu/SiO_2_. Science.

[27] Li S (2015). Kinetics study of hydrogenation of dimethyl oxalate over Cu/SiO_2_ catalyst. Ind. Eng. Chem. Res..

[28] Yan W (2019). X.Toward rational catalyst design for partial hydrogenation of dimethyl oxalate to methyl glycolate: a descriptor-based microkinetic analysis. Catal. Sci. Technol..

[29] Szabová L, Camellone M, Huang M, Matolín V, Fabris S (2010). Thermodynamic, electronic and structural properties of Cu/CeO_2_ surfaces and interfaces from first-principles DFT+U calculations. J. Phys. Chem. C..

[30] Yang Z, Xie L, Ma D, Wang G (2011). Origin of the high activity of the ceria-supported copper catalyst for H_2_O dissociation. J. Phys. Chem. C..

[31] Yang S (2018). Synergy between ceria oxygen vacancies and Cu nanoparticles facilitates the catalytic conversion of CO_2_ to CO under mild conditions. ACS Catal..

[32] Frenkel A, Hills C, Nuzzo R (2001). A view from the inside: complexity in the atomic scale ordering of supported metal nanoparticles. J. Phys. Chem. B.

[33] Cargnello M (2013). Control of metal nanocrystal size reveals metal-support interface role for ceria catalysts. Science.

[34] Goodman E (2019). Catalyst deactivation via decomposition into single atoms and the role of metal loading. Nat. Catal..

[35] Wen C, Cui Y, Chen X, Zong B, Dai W (2015). Reaction temperature controlled selective hydrogenation of dimethyl oxalate to methyl glycolate and ethylene glycol over copper-hydroxyapatite catalysts. Appl. Catal. B-Environ..

[36] Sun J (2018). Freezing copper as a noble metal–like catalyst for preliminary hydrogenation. Sci. Adv..

[37] Liu H, Jiang T, Han B, Liang S, Zhou Y (2009). Selective phenol hydrogenation to cyclohexanone over a dual supported Pd–Lewis acid catalyst. Science.

[38] Huang F (2019). Anchoring Cu_1_ species over nanodiamond-graphene for semi-hydrogenation of acetylene. Nat. Commun..

[39] Xu C (2018). Interfacing with silica boosts the catalysis of copper. Nat. Commun..

[40] Wang Y (2015). Insight into the Balancing Effect of active Cu species for hydrogenation of carbon-oxygen bonds. ACS Catal..

[41] Yin A, Guo X, Dai W, Fan K (2009). The nature of active copper species in Cu-HMS catalyst for hydrogenation of dimethyl oxalate to ethylene glycol: new insights on the synergetic effect between Cu^0^ and Cu^+^. J. Phys. Chem. C..

[42] Huang Y (2013). Silver-modulated SiO_2_-supported copper catalysts for selective hydrogenation of dimethyl oxalate to ethylene glycol. J. Catal..

[43] Yu H, Wei X, Li J, Gu S, Zhang S (2015). The XAFS beamline of SSRF. Nucl. Sci. Tech..

[44] Ravel B, Newville M (2005). Athena, Artemis, Hephaestus: data analysis for X-ray absorption spectroscopy using Ifeffit. J. Syn. Rad..

[45] Anderson, J. *Structure of Metallic Catalysts* (Academic Press, 1975).

[46] Perdew J (1992). Atoms, molecules, solids, and surfaces: applications of the generalized gradient approximation for exchange and correlation. Phys. Rev. B.

[47] Dudarev S, Botton G, Savrasov S, Humphreys C, Sutton A (1998). Electron-energy-loss spectra and the structural stability of nickel oxide: a LSDA+U study. Phys. Rev. B.

[48] Yang F (2011). CO oxidation on inverse CeO_x_/Cu(111) catalysts: High catalytic activity and ceria-promoted dissociation of O_2_. J. Am. Chem. Soc..

[49] Song W, Hensen E (2014). Mechanistic aspects of the water-gas shift reaction on isolated and clustered Au atoms on CeO_2_(110): A density functional theory study. ACS Catal..

[50] Rignanese G, DeVita A, Charlier J, Gonze X, Car R (2000). First-principles molecular-dynamics study of the (0001) α-quartz surface. Phys. Rev. B.

[51] Cui Y, Wang B, Wen C, Chen X, Dai W (2016). Investigation of activated-carbon-supported copper catalysts with unique catalytic performance in the hydrogenation of dimethyl oxalate to methyl glycolate. ChemCatChem.

[52] Yao D (2018). A high-performance nanoreactor for carbon–oxygen bond hydrogenation reactions achieved by the morphology of nanotube-assembled hollow spheres. ACS Catal..

[53] Yao D (2019). Balancing effect between adsorption and diffusion on catalytic performance inside hollow nanostructured catalyst. ACS Catal..

[54] Zhu Y (2014). The rise of calcination temperature enhances the performance of Cu catalysts: contributions of support. ACS catal..

[55] Ye R (2018). Synthesis of robust MOF-derived Cu/SiO_2_ catalyst with low copper loading via sol-gel method for the dimethyl oxalate hydrogenation reaction. ACS Catal..

[56] Cui G (2019). Low-temperature hydrogenation of dimethyl oxalate to ethylene glycol via ternary synergistic catalysis of Cu and acid-base sites. Appl. Catal. B: Environ..

[57] Yue H (2012). Hydrogenation of dimethyl oxalate to ethylene glycol on a Cu/SiO_2_/cordierite monolithic catalyst: enhanced internal mass transfer and stability. AIChE J..

