# Supplementary material for: Scalable synthesis of Cu clusters for remarkable selectivity control of intermediates in consecutive hydrogenation
Source: Nat Commun. 2023 Feb 27;14:1123. doi: 10.1038/s41467-023-36640-8 (PMC9970980; doi:10.1038/s41467-023-36640-8)
Supplement: Supplementary file 1 — Supplementary Information [file 41467_2023_36640_MOESM1_ESM.pdf]

Supplementary Information for

Scalable synthesis of Cu clusters for remarkable selectivity control of intermediates in consecutive hydrogenation

Dawei Yao[#1], Yue Wang[#1], Ying Li[1], Antai Li[1], Ziheng Zhen[1], Jing Lv[1], Fanfei Sun[2], Ruoou Yang[2, 3], Jun Luo[4], Zheng Jiang[2], Yong Wang*[5] and Xinbin Ma*[1]

[1] Key Laboratory for Green Chemical Technology of Ministry of Education, Collaborative Innovation Center of Chemical Science and Engineering, School of Chemical Engineering and Technology, Tianjin University, Tianjin 300072, China

[2] Shanghai Synchrotron Radiation Facility, Shanghai Advanced Research Institute, Chinese Academy of Sciences, Shanghai 201800, China

[3] State Key Laboratory of Materials Processing and Die & Mould Technology, School of Materials Science and Engineering, Huazhong University of Science and Technology, Wuhan, Hubei, 430074, China

[4] Institute for New Energy Materials, School of Materials, Tianjin University of Technology, Tianjin 300384, China

[5] Voiland School of Chemical Engineering and Bioengineering, Washington State University, Pullman, WA 99164, USA.

[#] Dr. Dawei Yao and Dr. Yue Wang contribute equally to this work.

Corresponding authors: xbma@tju.edu.cn, wang42@wsu.edu

Content

Supplementary Figure 1. Industrial process for producing methyl glycolate (MG), ethylene glycol (EG) and corresponding downstream products from syngas.

Supplementary Figure 2. Structure of lamellar Cu/SiO_2 and polyhedral CeO_2 precursors.

Supplementary Figure 3. TEM-EDS images of 5Cu-Stable after atomic thermal diffusion.

Supplementary Figure 4. Catalytic performance of 9Cu catalyst during different thermal treatment.

Supplementary Figure 5. Cu atomic diffusion between pellets.

Supplementary Figure 6. HAADF-STEM image of 5Cu-Stable and intensity line scans of the top atomic layers of CeO_2.

Supplementary Figure 7. HAADF-STEM image of 7Cu-Stable and intensity line scans of the top atomic layers of CeO_2.

Supplementary Figure 8. HAADF-STEM image of 7Cu-Stable with STEM-EELS analysis.

Supplementary Figure 9. Structural information of Cu clusters in 5Cu and 7Cu that are thermal treated under N_2.

Supplementary Figure 10. STEM-EDS images of 5Cu-Stable catalyst.

Supplementary Figure 11. Chemical properties of Cu species in 9Cu catalyst during atomic diffusion.

Supplementary Figure 12-13. The optimized structure of Cu_n cluster on CeO_2 (100) and the potential anchoring position for Cu_{n+1} atom.

Supplementary Figure 14-15. The optimized structure of Cu_n cluster on CeO_2 (110) and the potential anchoring position for Cu_{n+1} atom.

Supplementary Figure 16. Chemical properties of xCu-Stable catalysts.

Supplementary Figure 17. High-resolution STEM-EDX images of the 9Cu-Stable catalysts.

Supplementary Figure 18. High-resolution STEM-EDX images of the 11Cu-Stable catalysts.

Supplementary Figure 19. Pictures of ~1 kg of the 5Cu catalyst produced in one batch.

Supplementary Figure 20. MG TPD-MS of Cu-Stable catalysts with different Cu domain sizes.

Supplementary Figure 21. Adsorption energies of MG on CeO_2 supported Cu species with different Cu atoms numbers.

Supplementary Figure 22. Free energy profiles for MG+H* on CeO_2 (110) supported Cu species with different Cu atom numbers.

Supplementary Figure 23. Adsorption energies of ethylene on CeO_2 supported Cu species with different Cu atom numbers.

Supplementary Figure 24. Free energy profiles for H_2 dissociation on CeO_2 supported Cu species with different Cu atom numbers.

Supplementary Table 1. Cu loading, crystalline size and catalytic performance of the lamellar Cu/SiO_2 and CeO_2 precursors

Supplementary Table 2. Cu-K adsorption energy and EXAFS fitting results of 9Cu catalysts in different stages

Supplementary Table 3. EXAFS fitting results of xCu-Stable catalysts.

Supplementary Table 4. Catalytic performance of reported Cu-based catalysts for DMO hydrogenation to MG.

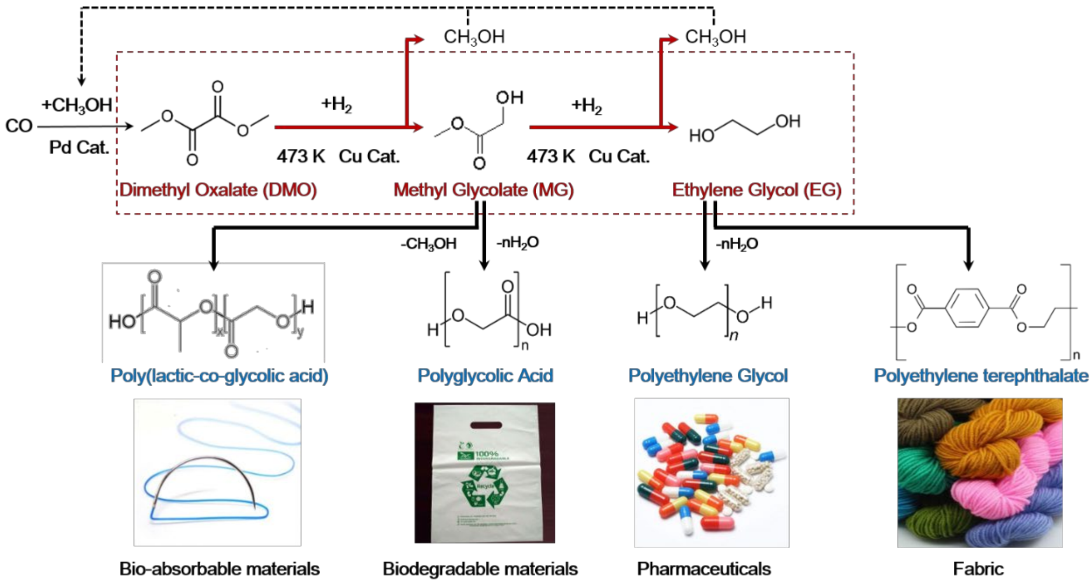

Supplementary Figure 1. Industrial process for producing methyl glycolate (MG), ethylene glycol (EG) and corresponding downstream products from syngas. This process consisted of coupling of CO with methanol to form dimethyl oxalate (DMO) and subsequent hydrogenation to yield MG and EG. The by-product methanol could be recycled to the coupling process. EG has a wide range of application in various fields, such as the antifreeze and coolant in automobiles, the deicing fluid for windshields and aircraft, and the feedstock for polyester fibers and resins synthesis[1]. MG is the important intermediate for producing polyglycolic acid and poly(lactic-co-glycolic acid), which are biodegradable, biocompatible and thermoplastic polymers. With the growing environmental concerns, polyglycolic acid and poly(lactic-co-glycolic acid) have attracted much attention due to their advantages in producing degradable plastics and bioresorbable surgical materials.

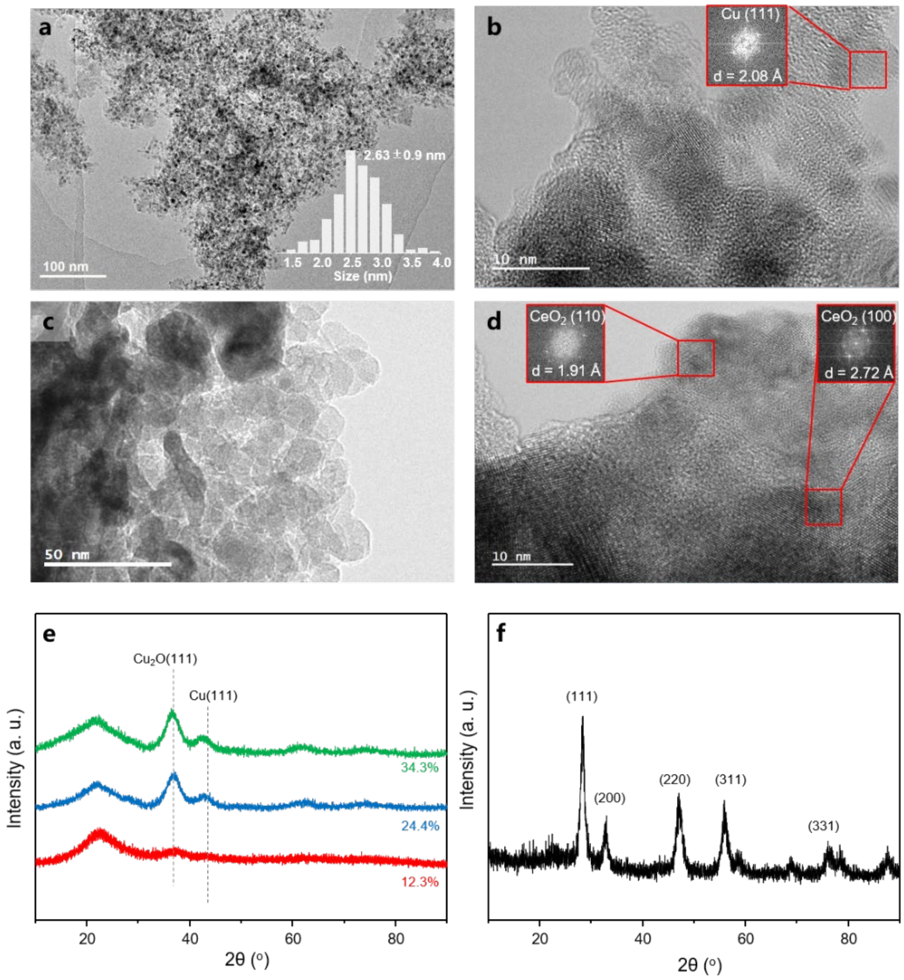

Supplementary Figure 2. Structure of lamellar Cu/SiO_2 and polyhedral CeO_2 precursors. a-b, TEM images and corresponding FFT images of the reduced lamellar Cu/SiO_2. **c-d,** TEM images of polyhedral CeO_2 obtained by calcination of cerium nitrate. **e,** XRD patterns of the reduced lamellar Cu/SiO_2 with different copper loadings. The corresponding crystalline sizes of Cu and Cu_2O are listed in Supplementary Table 1, which are calculated according to the peaks at 2θ of 43.4° and 37.0° by Scherrer equation. Benefitting from the unique lamellar structure of copper phyllosilicate[2], the size of Cu NPs could be easily restrained to ~3 nm. **f,** XRD patterns of the polyhedral CeO_2. Both of surface facets of (110) and (100) are exposed in the polyhedral CeO_2, as well as (111) facet shown in the XRD pattern.

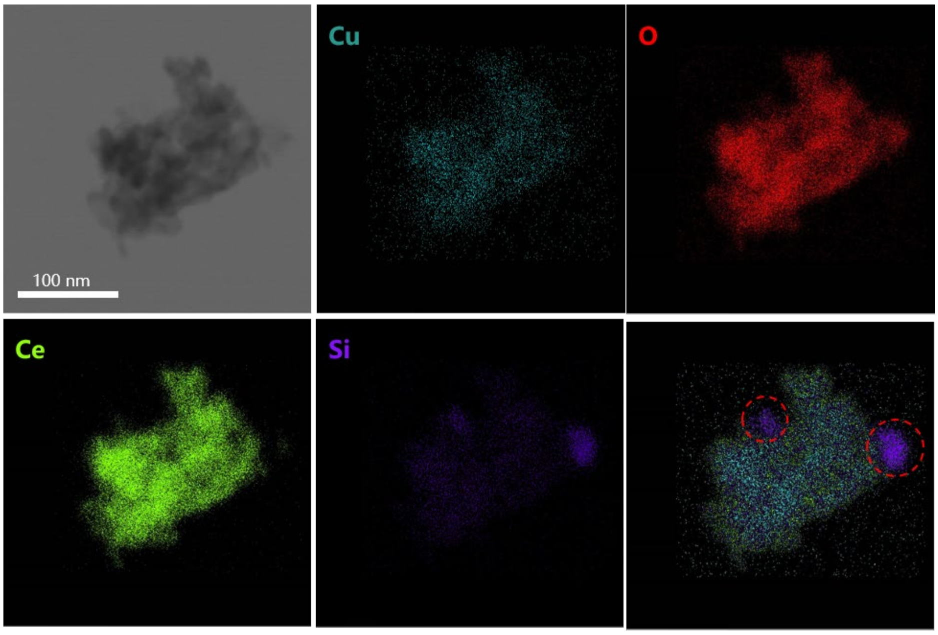

Supplementary Figure 3. TEM-EDS images of 5Cu-Stable after atomic thermal diffusion. The lamellar structured copper silicate precursors were first mixed with CeO_2 at a weight ratio of 1:3.6, followed by reduction in pure H_2 at 300 °C for 4 h and further treatment in pure H_2 at 200 °C for 100 h. As a result, most Cu species appeared on CeO_2 rather than SiO_2, verifying the atomic diffusion of Cu.

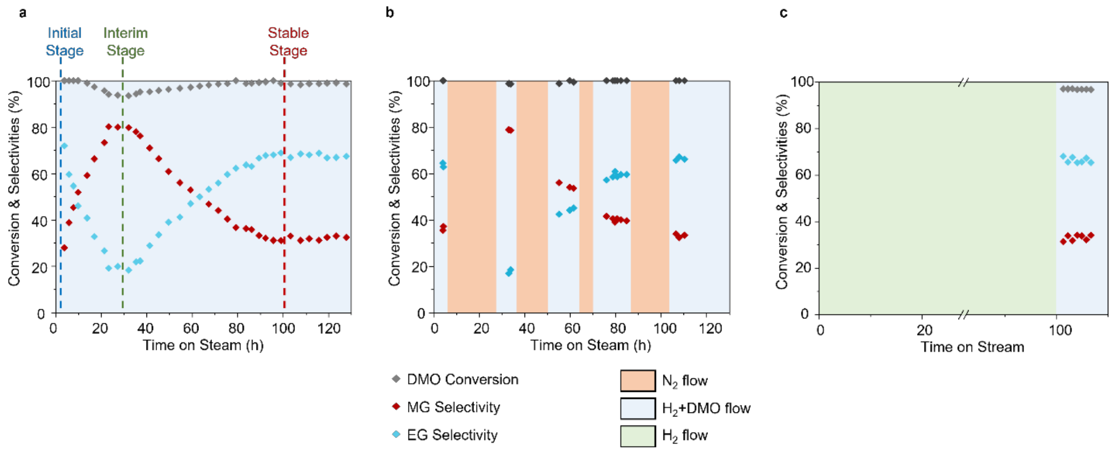

Supplementary Figure 4. Catalytic performance of 9Cu catalyst during different thermal treatment. a, Catalytic performance under the continuous reactants feed flow (H_2 and vaporized DMO mixed with a molar ratio of 80). **b,** Catalytic performance under randomly switched H_2+DMO flow and N_2 flow. The blue background indicates the catalyst was treated in reactant flow and the orange background indicates the catalyst was treated in N_2 flow. **c,** Catalytic performance after 100 h thermal treatment under H_2 flow. The green background indicates the catalyst was treated in H_2 flow and the blue background indicates the catalyst was treated in reactant flow. Gary diamonds: DMO conversion. Blue diamonds: EG selectivity. Red diamonds: MG selectivity. Reaction conditions: 200 °C, $WLHSV_{Cu}$=6 h^{-1}, H_2/DMO=80. The flow rates of the different gases for treatment were kept identical. It can be seen that the catalytic performances under different gas feeds are very similar, indicating that the Cu atomic thermal diffusion process is not obviously influenced by the treatment gases (H_2, N_2 or the reactants).

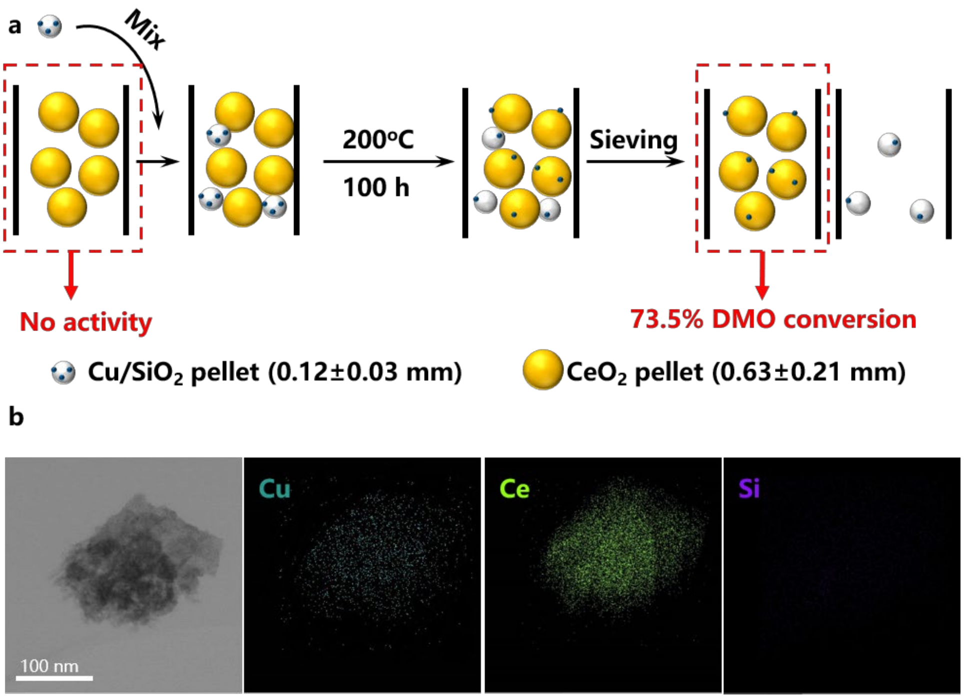

Supplementary Figure 5. Cu atomic diffusion between pellets. a, Illustration of the separation principle of pellets mixture and their respective activity in DMO hydrogenation. The starting materials of Cu/SiO_2 and CeO_2 pellets are the same as those for 5Cu catalyst preparation. After shaped to different sizes, the two types of pellets were reduced separately under 300°C for 4 h and then mixed at a weight ratio of 3.6 (the same as 5Cu catalyst). Subsequently, the mixed pellets were treated at 200°C under H_2 gas. After treatment, the CeO_2 pellets were collected by sieving the mixed pellets. Both the sieved CeO_2 pellets after low-temperature thermal treatment and the original ones before mixing were evaluated in DMO hydrogenation. Reaction conditions are: 200 °C, $WLHSV_{Cat.}$=0.24 h^{-1}, H_2/DMO=80. Over sieved CeO_2 pellet, the conversion of DMO is 73.5% and the MG selectivity is 100%, while the original ones showed no activity in DMO hydrogenation. **b**, TEM-EDS images of sieved CeO_2 pellets after low-temperature thermal treatment. Copper species could be detected on the CeO_2 support.

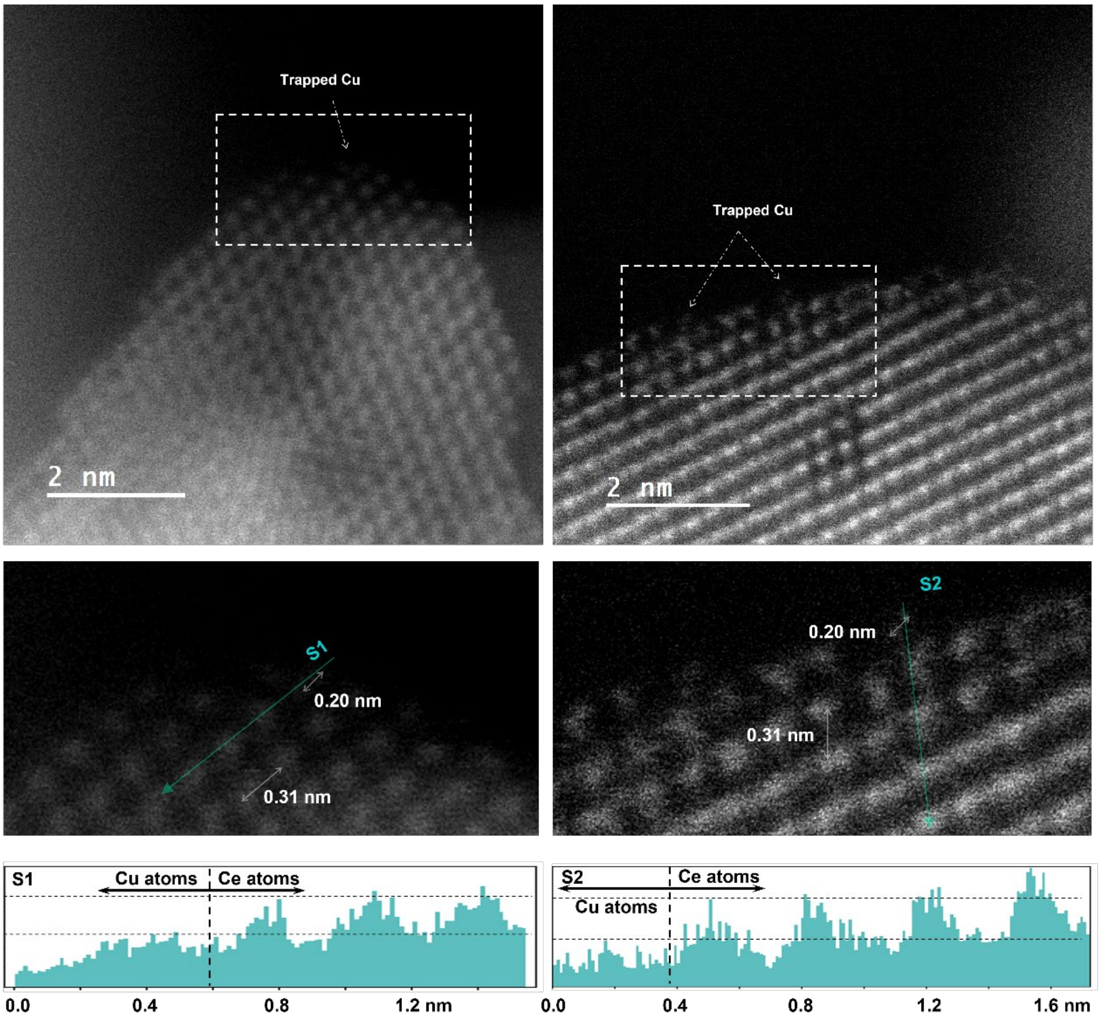

Supplementary Figure 6. HAADF-STEM image of 5Cu-Stable and intensity line scans of the top atomic layers of CeO_2.

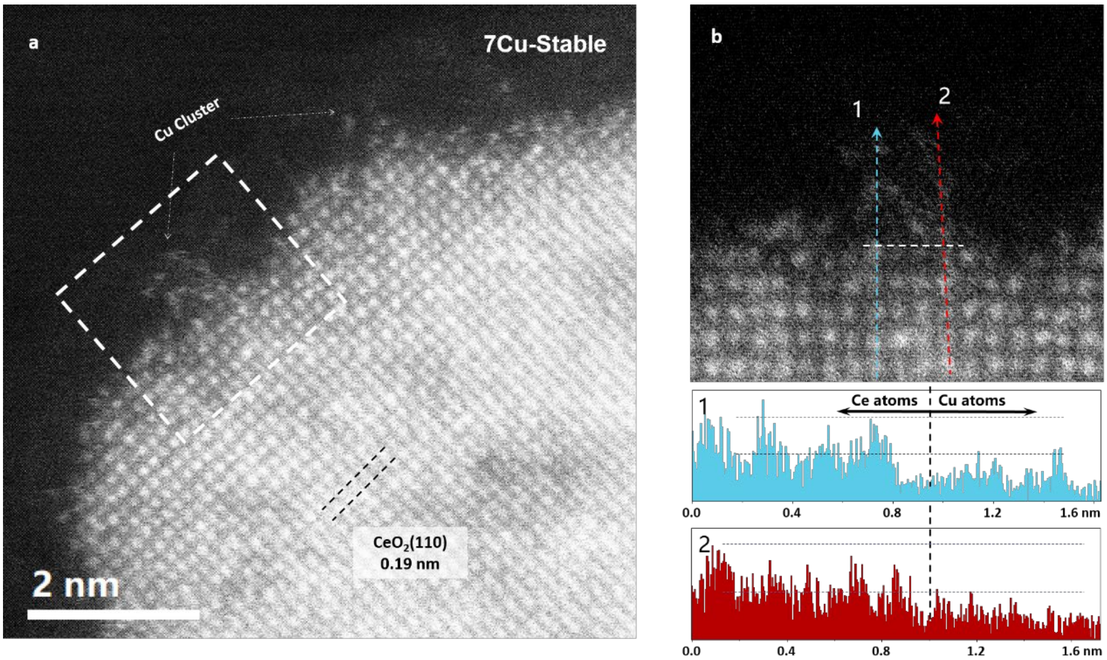

Supplementary Figure 7. HAADF-STEM image of 7Cu-Stable and intensity line scans of the top atomic layers of CeO_2. a, HAADF-STEM image of 7Cu-Stable. The top five layers of CeO_2 edge (17×5 atoms) surface was magnified to show the detailed atomic arrangement in b. **b,** Intensity line scans 1&2 of the top atomic layers of CeO_2 crystal and the corresponding intensity. The interface between Cu and Ce can be demonstrated according to the intensity line scans.

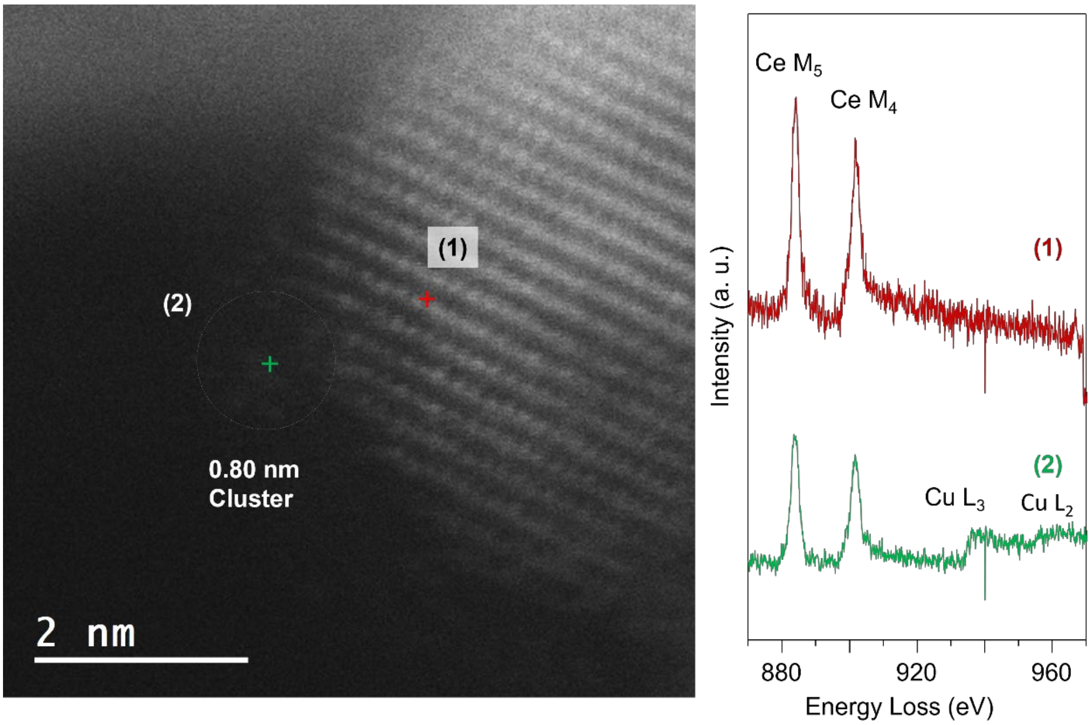

Supplementary Figure 8. HAADF-STEM image of 7Cu-Stable with STEM-EELS analysis.

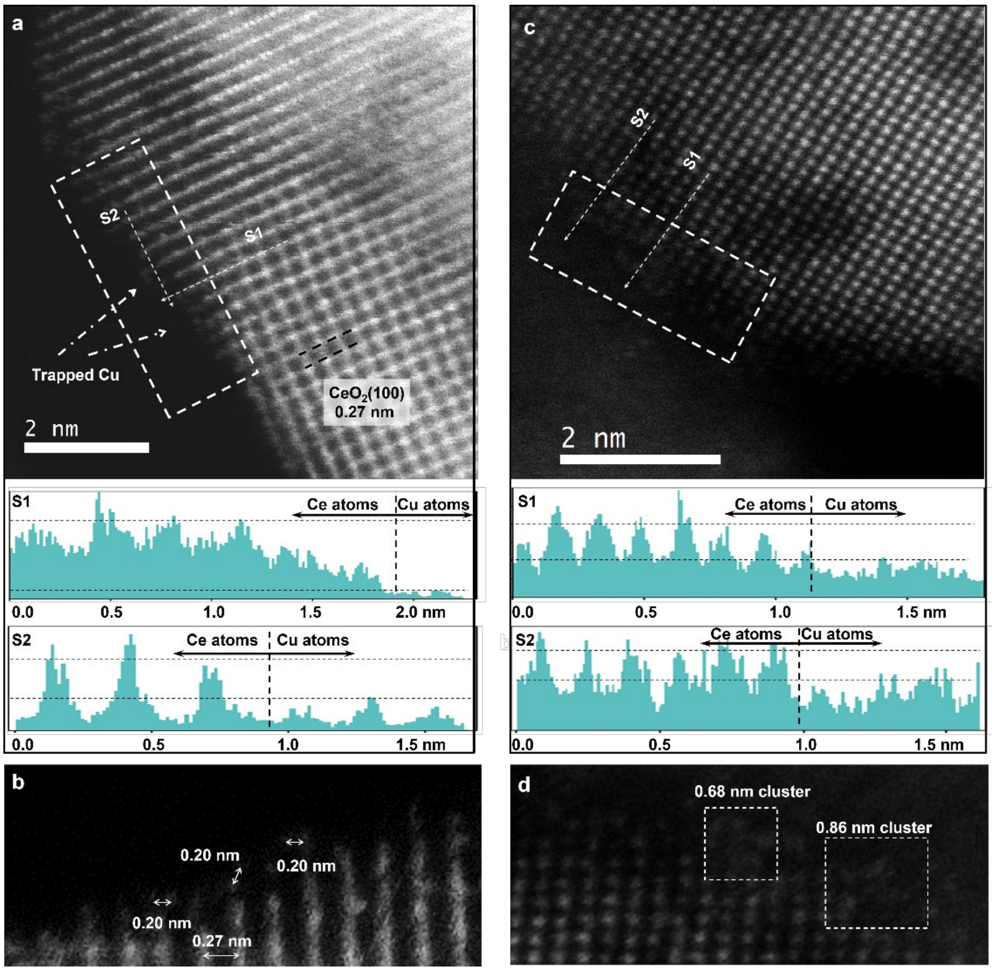

Supplementary Figure 9. Structural information of Cu clusters in 5Cu and 7Cu that are thermally treated under N_2 at 200 °C for 100 h. a, HAADF-STEM image of N_2 thermal treated 5Cu and intensity line scans from two directions (S1 and S2) of the top atomic layers of CeO_2. The top three layers of CeO_2 edge were magnified as shown in **b** with measured atom distances. The distance between the darker atoms and the brighter atoms are 0.20 nm and 0.27 nm respectively, corresponding to the interatomic distance of Cu-Cu and CeO_2 (100). **c,** HAADF-STEM image of N_2 thermal treated 7Cu and intensity line scans from two directions (S1 and S2) of the top atomic layers of CeO_2. The top several layers of CeO_2 with edge was magnified as shown in **d**.

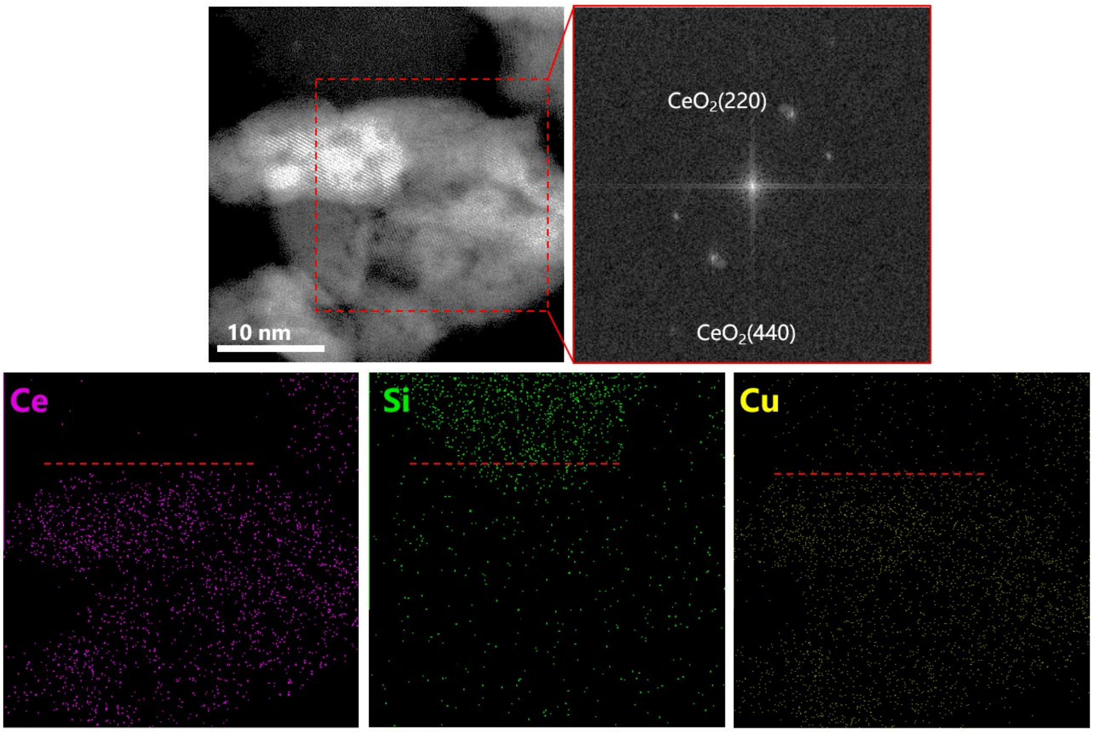

Supplementary Figure 10. STEM-EDS images of 5Cu-Stable catalyst. The Cu distribution on SiO_2 and CeO_2 demonstrates most of Cu species appears on CeO_2 rather than SiO_2. The absence of lattice fringes of any Cu species in the corresponding fast Fourier transform image indicates the high dispersion of Cu atoms.

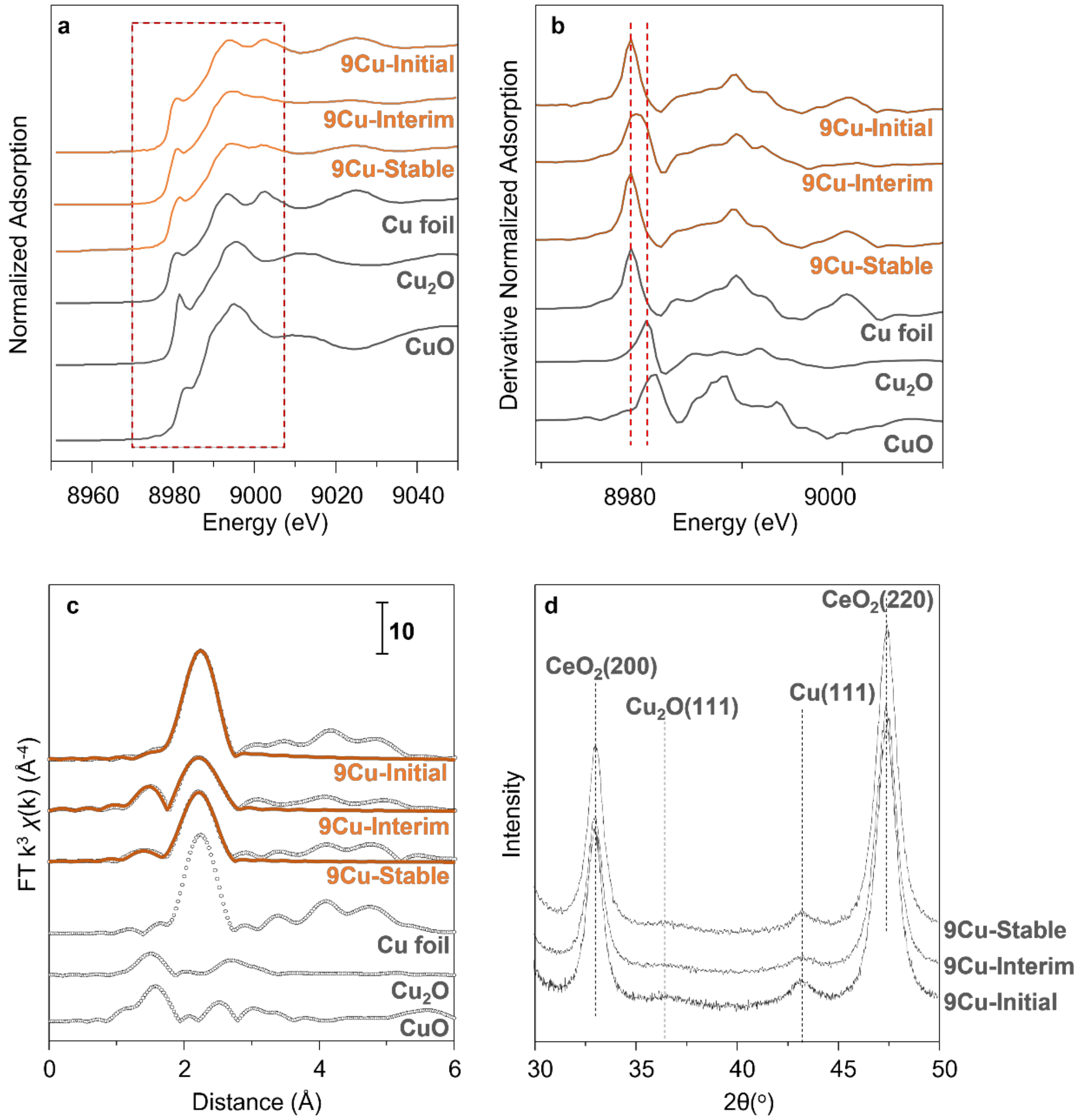

Supplementary Figure 11. Chemical properties of Cu species in 9Cu catalyst during atomic diffusion. a, Cu K XANES spectra and **b,** first derivative of 9Cu catalysts with different treatment stages according to Supplementary Figure 3, the Cu K adsorption energies are listed in Supplementary Table 2. **c,** Cu K edge R space EXAFS spectra of 9Cu catalysts with different treatment stages. The dots are original data and lines are fitted by FEFF code from the crystal structures previously reported (JCPDS card#: 4-836, 5-667 and 41-254 for Cu, Cu_2O and CuO respectively). The fitted structural information of Cu is shown in Supplementary Table 2. The Cu-Cu

coordination number first decreased to 5.43 (9Cu-Interim) then rose back to 8.18 (9Cu-Stable) during the reaction, indicating the size of Cu species decreases at first, then increases back and the Cu NPs are regenerated eventually during the atomic thermal diffusion. **d**, XRD patterns of 9Cu catalysts with different treatment stages. The change trend of Cu(111) diffraction peak is consisted with the corresponding variation of Cu-Cu coordination number. It could be deduced from the variation of chemical state, Cu-Cu coordination number and crystalline size of the Cu species, that this low-temperature atomic diffusion process contains two steps. The first step is that Cu atoms are emitted from Cu NPs and then trapped on CeO_2 to form stable SAs Cu. The second step is the excess Cu species continue to be emitted and orderly anchored around the Cu species on CeO_2 to form Cu clusters or NPs.

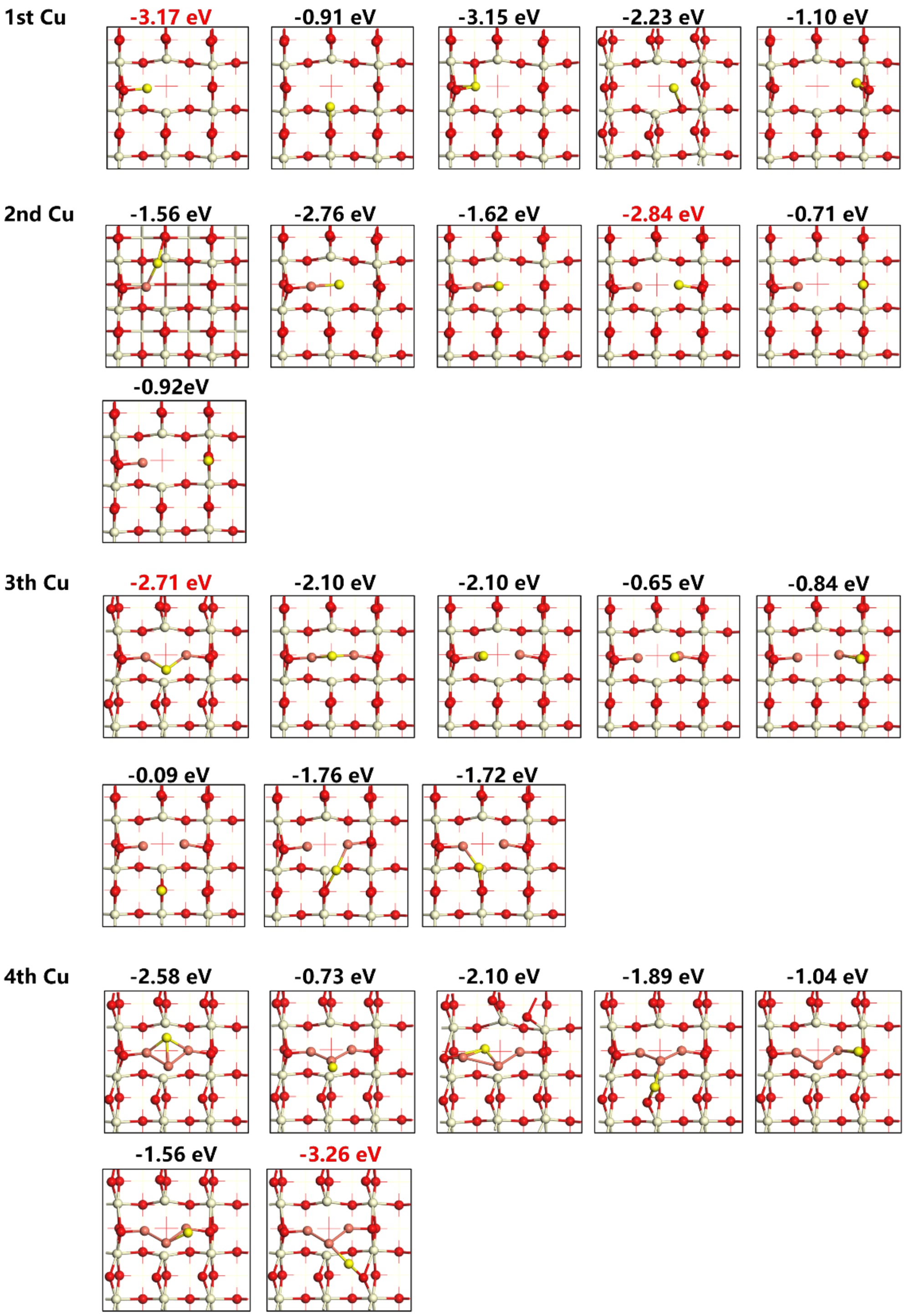

Supplementary Figure 12. The optimized structure of Cu_n cluster on CeO_2 (100) and the potential anchoring position for Cu_{n+1} atom. This figure shows the anchor of 1st~4th Cu atom.

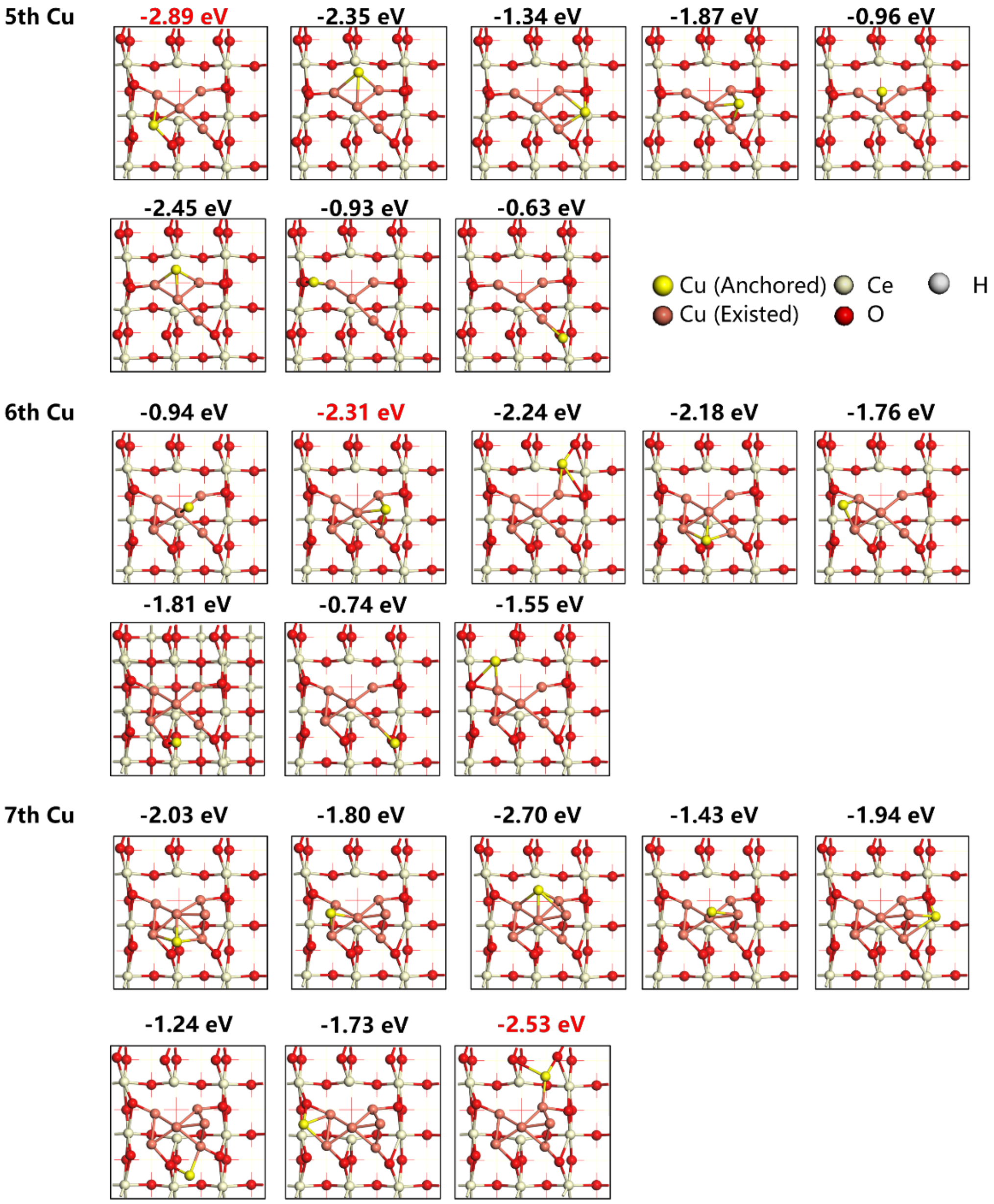

Supplementary Figure 13. The optimized structure of Cu_n cluster on CeO_2 (100) and the potential anchoring position for Cu_{n+1} atom. This figure shows the anchor of 5th~7th Cu atom.

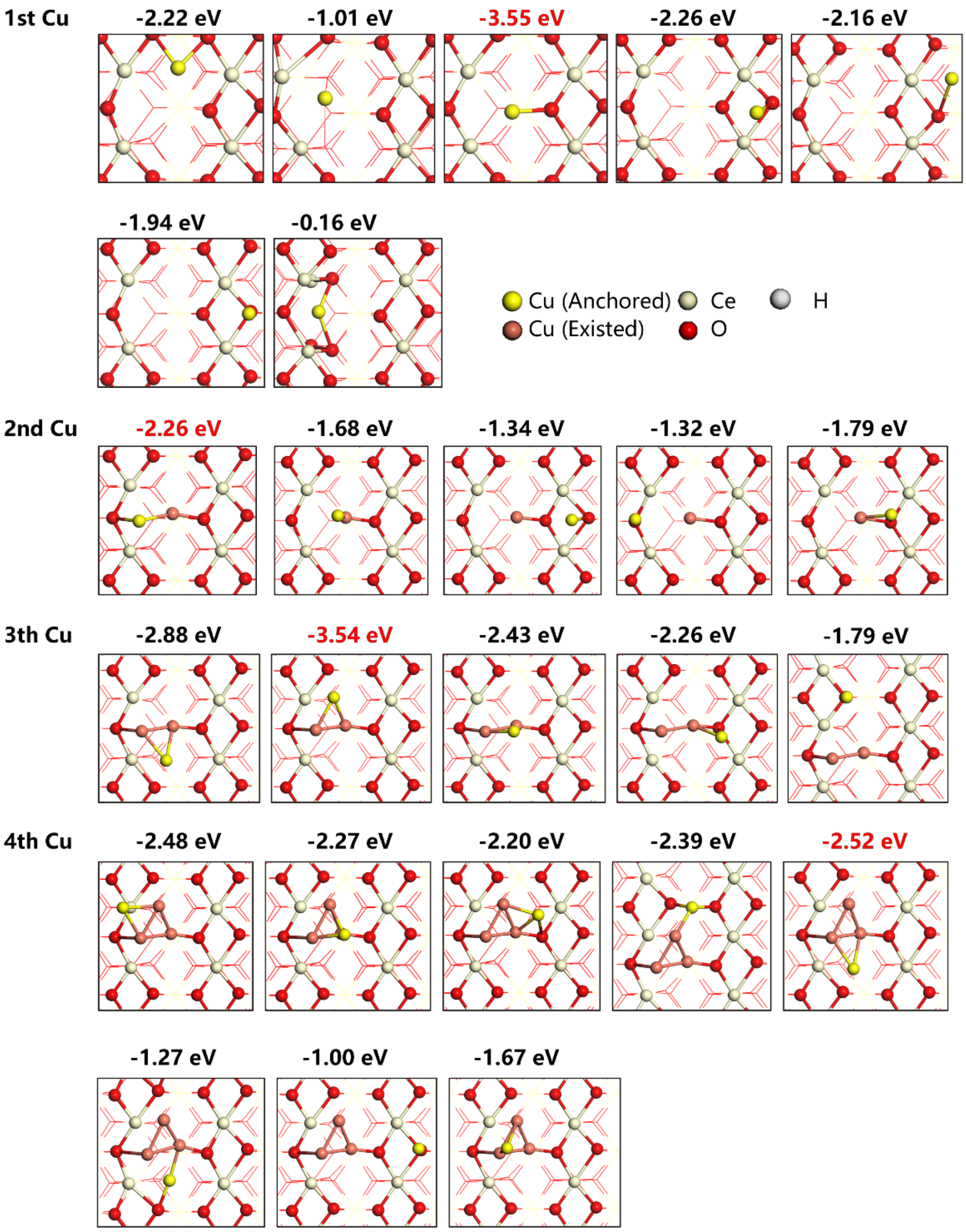

Supplementary Figure 14. The optimized structure of Cu_n cluster on CeO_2 (110) and the potential anchoring position for Cu_{n+1} atom. This figure shows the anchor of 1st~4th Cu atom.

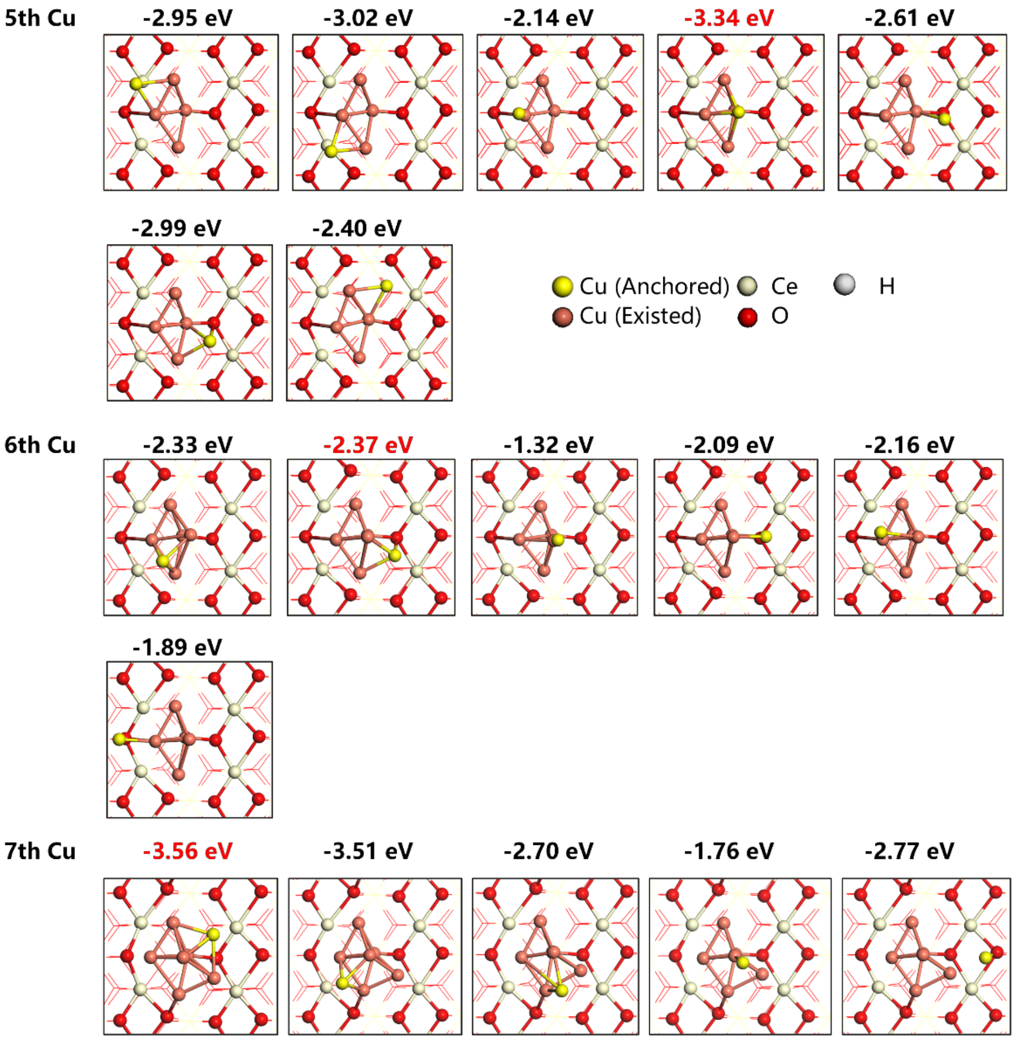

Supplementary Figure 15. The optimized structure of Cu_n cluster on CeO_2 (110) and the potential anchoring position for Cu_{n+1} atom. This figure shows the anchor of 5th~7th Cu atom.

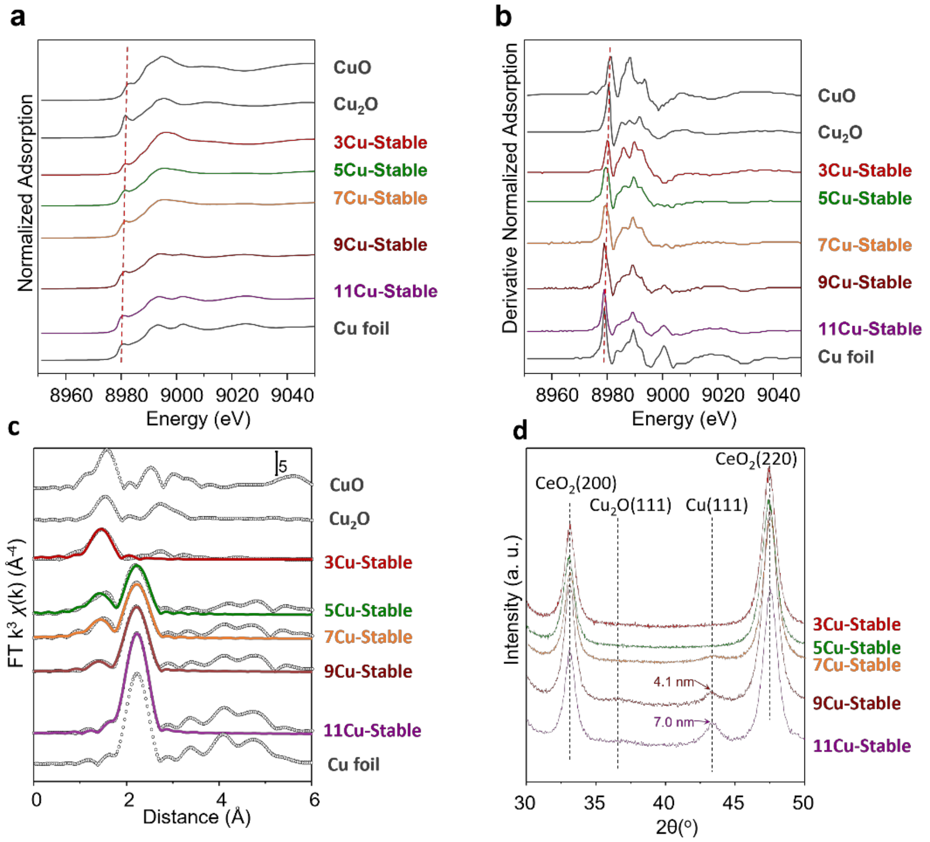

Supplementary Figure 16. Chemical properties of xCu-Stable catalysts. a, Cu K edge XANES spectra of xCu-Stable catalysts; **b,** Derivative of XANES spectra. According to the derivative profile, the pre-edge of XANES of xCu-Stable could be precisely determined. **c,** EXAFS R space spectra of xCu-stable catalysts. The dots are original data and lines are fitted results. The fitted CN of Cu are shown in Supplementary Table 3. **d,** XRD patterns of the xCu-stable catalysts. The crystalline size of Cu is calculated according to Cu (111) diffraction peak at 42.2° by Scherrer equation. Additionally, the high dispersion of Cu SAs and clusters in xCu-Stable (x=3, 5, 7) were further confirmed by the absence of diffraction peaks of Cu species in XRD patterns. As the Cu loading is increased, the Cu (111) diffraction peak, Cu-Cu (2.24 Å) peaks, as well as the Cu-Cu CN increased, indicating the gradual raising size of Cu species.

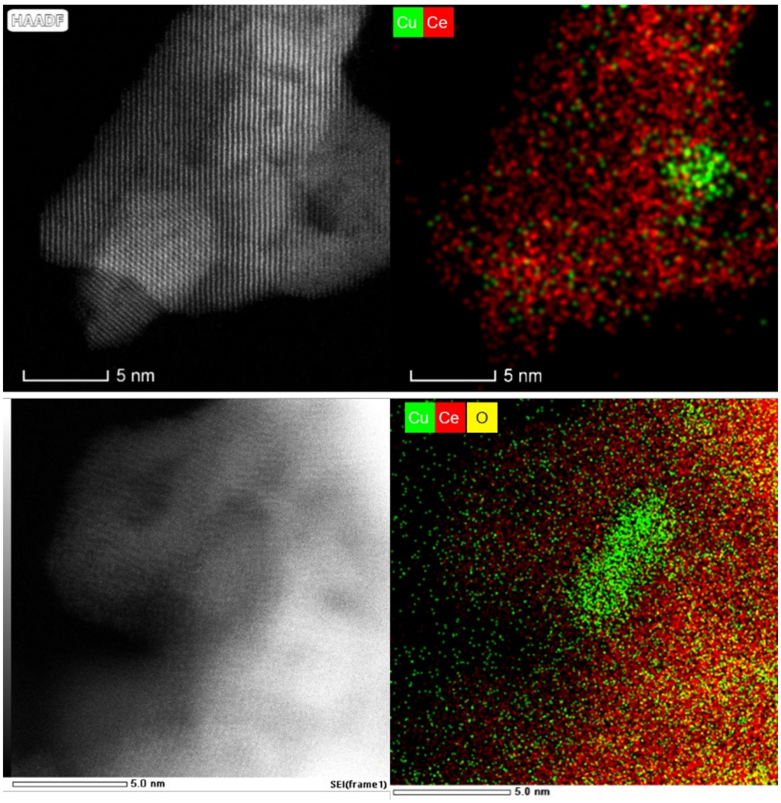

Supplementary Figure 17. High-resolution STEM-EDX images of the 9Cu-Stable catalysts.

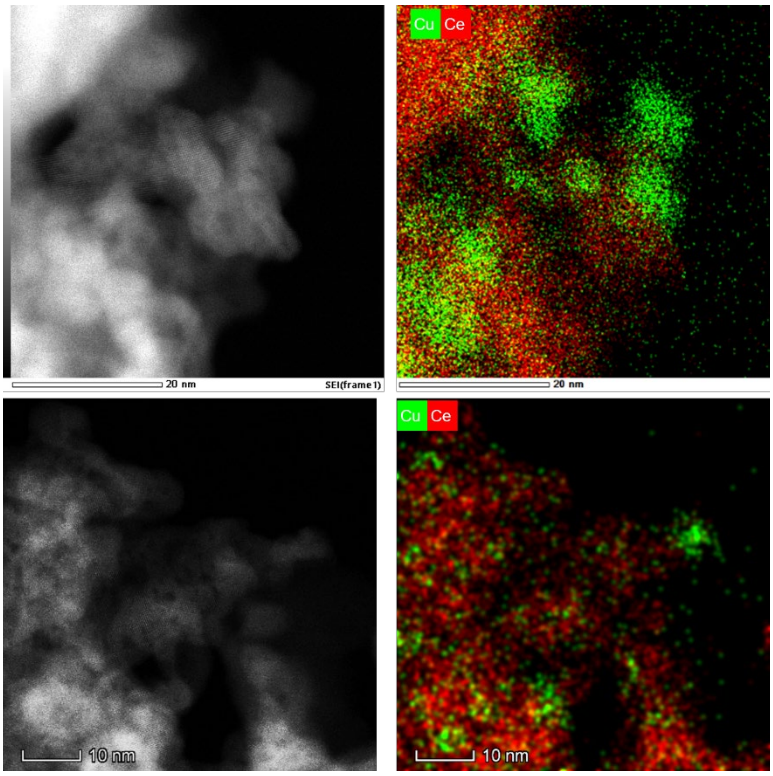

Supplementary Figure 18. High-resolution STEM-EDX images of the 11Cu-Stable catalysts.

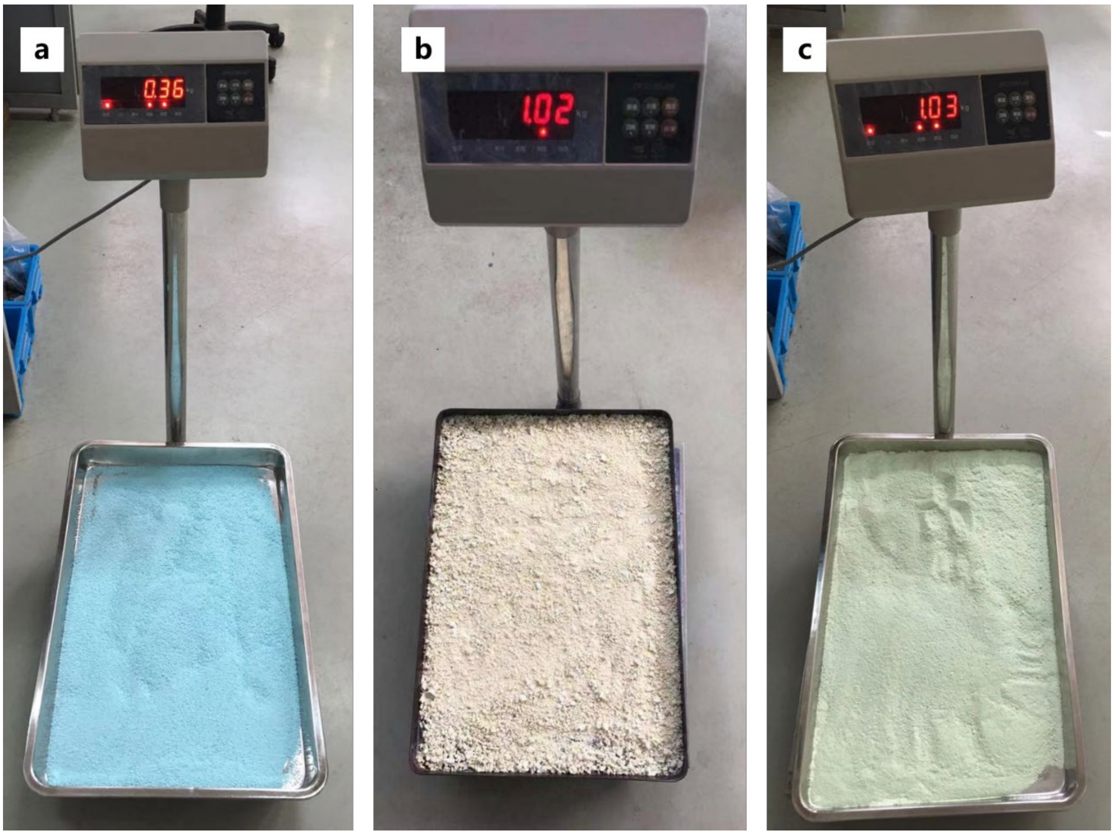

Supplementary Figure 19. Pictures of ~1 kg of the 5Cu catalyst produced in one batch. a, Lamellar-structured copper silicate precursor, which could be synthesized by ammonia evaporation method in a large scale[2]. **b**, CeO_2 precursor, obtained by calcination of cerium nitrate hexahydrate air at 350 ˚C for 2 h. The procedure of large-scale synthesis of Cu catalysts is same to the lab-scale synthesis. For example, 0.36 kg copper silicate powder and 1 kg CeO_2 are physically mixed at a weight ratio of 1:3.6 and grounded for 15 min to obtain the green powder as shown in **c**. **c,** As-prepared 5Cu catalysts. The unit of the displayed number is kilogram.

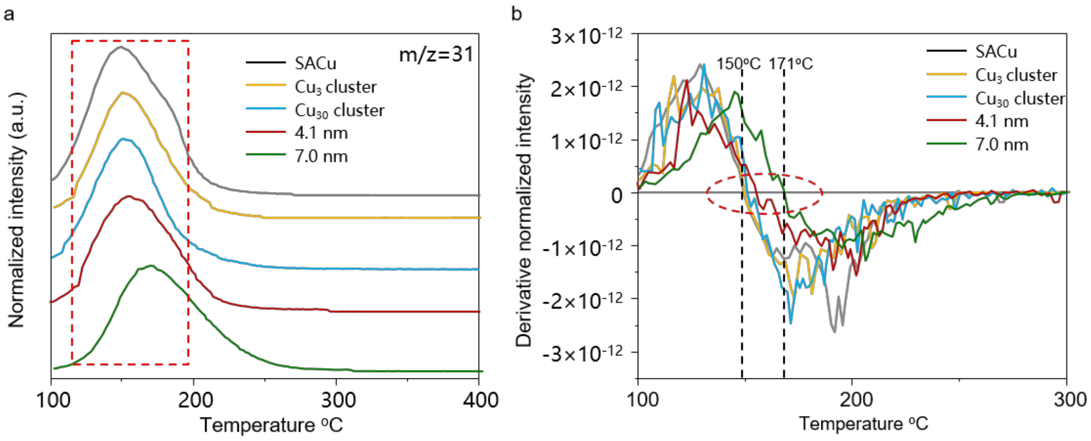

Supplementary Figure 20. MG TPD-MS of Cu-Stable catalysts with different Cu domain sizes. a, MG TPD-MS profiles. The methoxyl group signal (m/z=31) is measured to demonstrate the MG desorption behavior. Due to MG can not be decomposed under low temperature, all the methoxyl group is generated by the ionization of MG inside MS. The intensity of MS signal is normalized according to the amount of total surface Cu species. **b,** Derivative of normalized intensity of MG TPD-MS profiles. According to the derivative profiles, the peak position in MG TPD-MS could be precisely determined. It could be seen the MG desorption peak shifts from 150°C to 171°C with the increased size of Cu species from single atom to 7 nm, indicating that the adsorption of MG is stronger on larger Cu species.

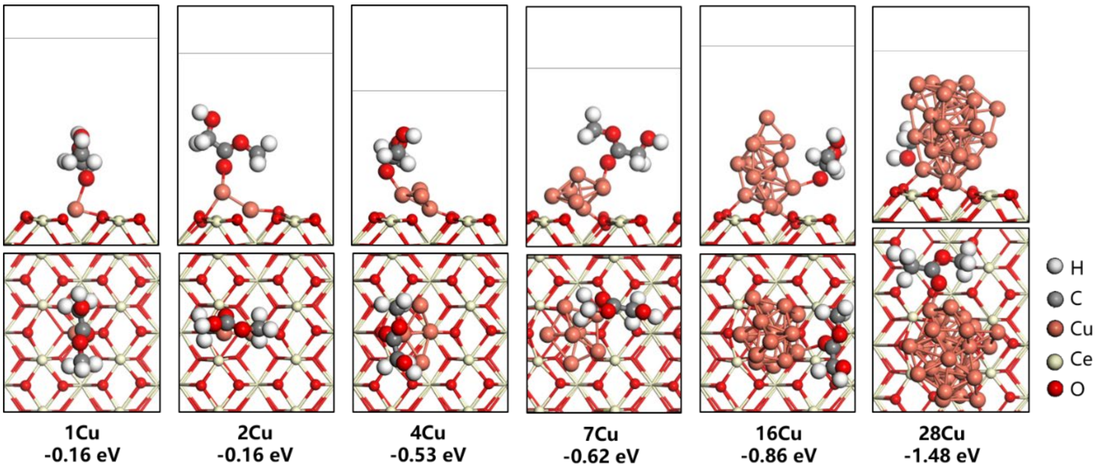

Supplementary Figure 21. Adsorption energies of MG on CeO_2 supported Cu species with different Cu atoms numbers. The most stable adsorption site of MG is near the interface between the Cu cluster and support, where the O end of C=O group connected with the Cu species at the perimeter, correspond to theoretical study reported by previous works[3]. These Cu species with electronic structure of $Cu^{\delta+}$ at the perimeter are responsible to absorb and activate methoxy and acyl species[4, 5]. For the Cu SA and Cu_2 cluster, the MG adsorption energies are both -0.16 eV, indicating that MG is easily to desorb instead of being further hydrogenated to EG. Furthermore, the adsorption energies on Cu_n clusters gradually decrease from -0.53 eV to -1.48 eV with the increased Cu number from 4 to 28, indicating that the stability of MG is increased on Cu species with higher atom numbers.

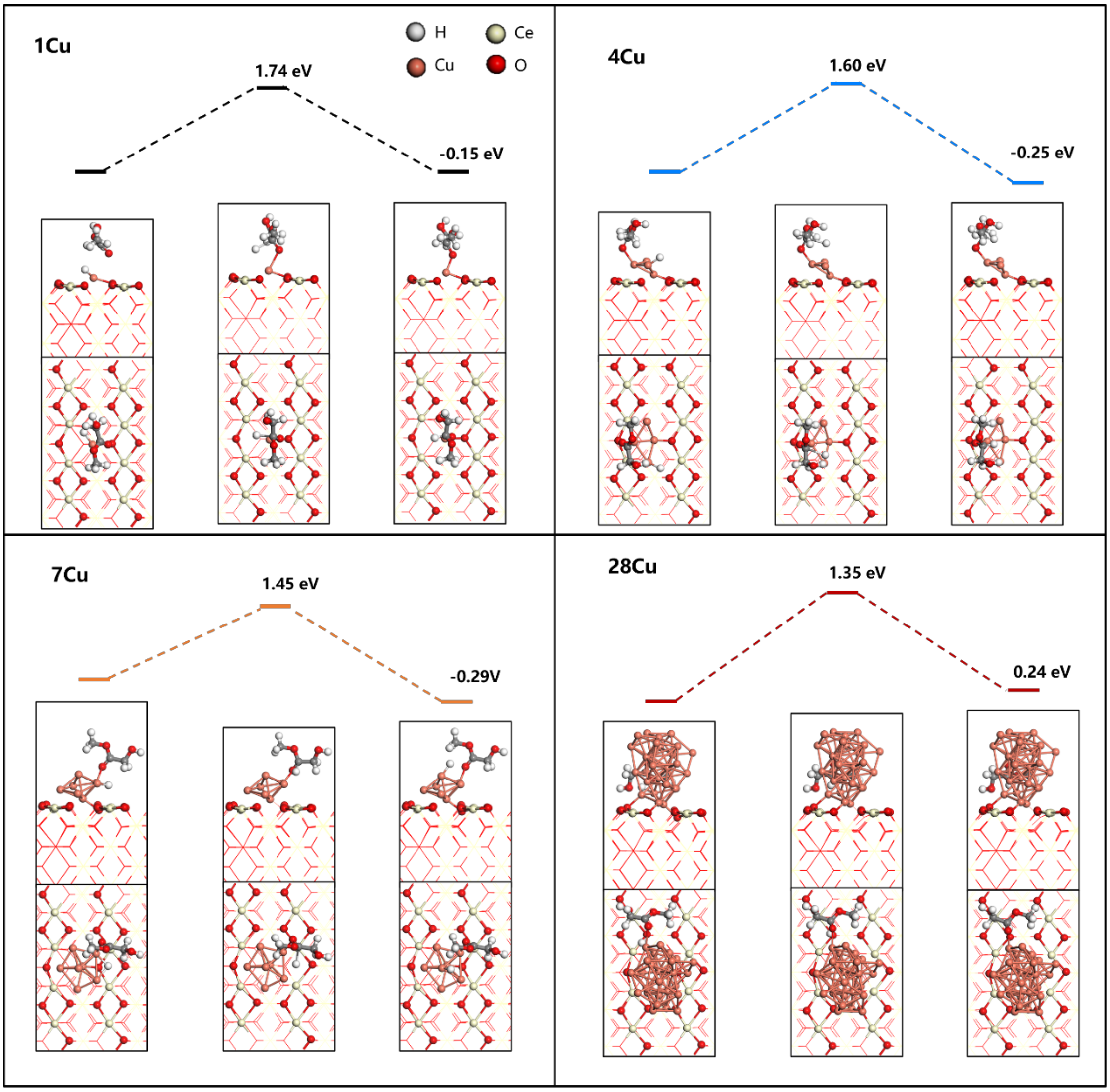

Supplementary Figure 22. Free energy profiles for MG+H* on CeO_2 (110) supported Cu species with different Cu atom numbers.

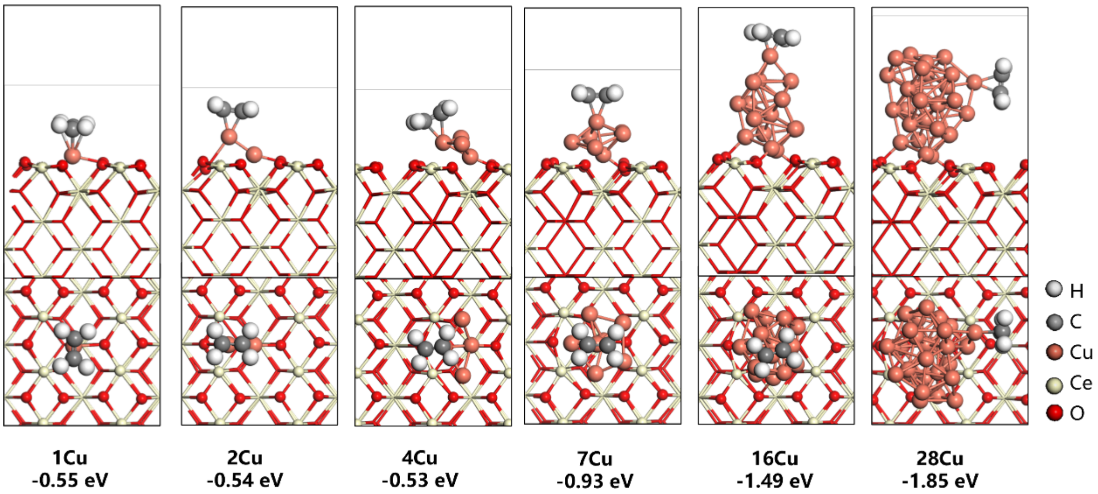

Supplementary Figure 23. Adsorption energies of ethylene on CeO_2 supported Cu species with different Cu atom numbers. The adsorption energies of ethylene on Cu species gradually decrease from about -0.55 eV to -1.85 eV with the increased Cu number from 1 to 28, indicating that the stability of ethylene is increased on Cu species with higher atom numbers.

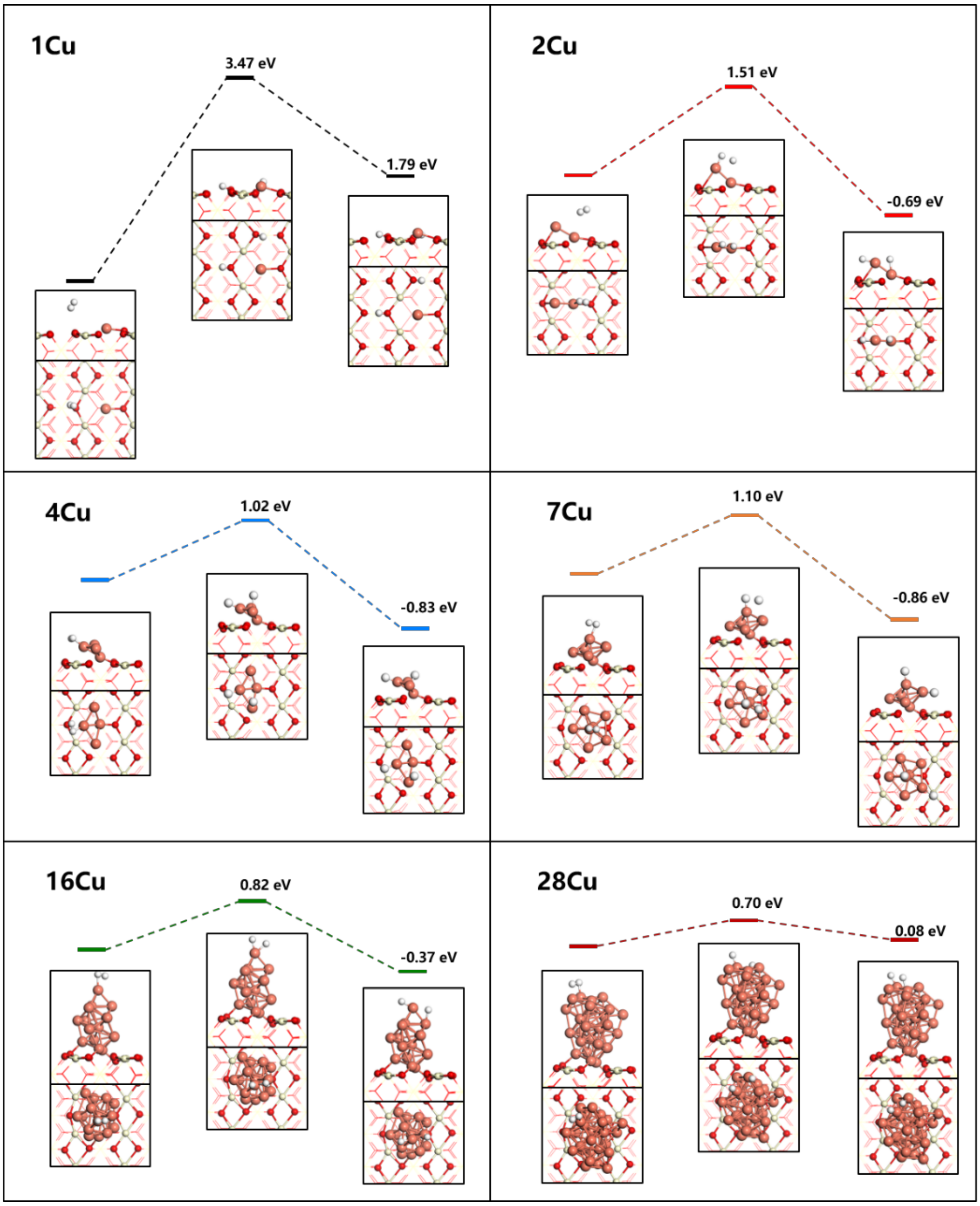

Supplementary Figure 24. Free energy profiles for H_2 dissociation on CeO_2 supported Cu species with different Cu atom numbers.

Supplementary Table 1. Cu loading, crystalline size and catalytic performance of the lamellar Cu/SiO_2 and CeO_2 precursors

Cu loading in Precursors[a] (wt. %)	Crystalline size (nm)[b]		DMO conversion (%)[c]	MG selectivity (%)[c]	EG selectivity (%)[c]	Cu loading of the catalyst
	Cu_2O	Cu				
12.3%Cu/SiO_2[a]	2.7	-[d]	100	3.4	94.6	2.7% (3Cu)
24.4%Cu/SiO_2	2.9	2.9	100	5.7	92.4	5.4% (5Cu)
34.3%Cu/SiO_2	2.9	3.0	100	4.5	90.3	7.5% (7Cu)
CeO_2	-	-	0	-	-	

[a] Cu loading was measured by ICP-OES. [b]Calculated by XRD patterns. [c]Catalytic performance of Cu/SiO_2 precursors, reaction conditions: 200 °C, $WLHSV_{Cu}$= 6 h^{-1}, H_2/DMO=80. Trace byproducts were detected including ethanol and C_3–C_4 diols. [d]Not detected in the XRD pattern.

Supplementary Table 2. Cu-K adsorption energy and EXAFS fitting results of 9Cu catalysts in the different stages

Catalyst	Cu-K adsorption energy (eV)	Path	R(Å)[a]	CN[b]	σ^2(10^{-3}Å^2)[c]	ΔE_0(eV)[d]	R-factor
9Cu-Initial	8979.0	Cu-Cu	2.54±0.02	9.6±0.9	9.1	4.14	0.002
		Cu-O	-[e]	-	-		
9Cu-Interim	8979.5	Cu-Cu	2.55±0.03	5.4±0.5	8.4	5.27	0.009
		Cu-O	1.85±0.02	1.0±0.1	0.8		
9Cu-Stable	8979.0	Cu-Cu	2.53±0.02	8.2±0.8	9.3	3.10	0.009
		Cu-O	1.82±0.02	0.4±0.1	7.8		
Cu[f]	8979.0	Cu-Cu	2.56	12			
		Cu-O					
Cu_2O[f]	8980.8	Cu-Cu	3.70	8			
		Cu-O	1.85	4			
CuO[f]	8983.8	Cu-Cu	2.91	4			
		Cu-O	1.91	2			
		Cu-O	1.99	2			

[a]R is interatomic distance. [b]CN is coordination number. [c]σ^2 is Debye-Waller factor, a measure of thermal and static disorder in absorber scatter distances. [d]ΔE_0 is edge energy shift. [e]No reliable fitted values can be obtained. [f]EXAFS data (R and CN) were calculated by FEFF code from the reported crystal structures[32] (JCPDS card#: 4-836, 5-667 and 41-254 for Cu, Cu_2O, and CuO, respectively)

Supplementary Table 3. EXAFS fitting results of the xCu-Stable catalysts.

Catalyst	Cu-K adsorption energy (eV)	Path	R(Å)[a]	CN[b]	σ^2(10^{-3}Å^2)[c]	ΔE_0(eV)[d]	R-factor
3Cu-Stable	8980.5	Cu-Cu	-[e]	-	-	3.44	0.020
		Cu-O	1.85±0.02	1.3±0.1	7.6		
5Cu-Stable	8979.6	Cu-Cu	2.53±0.02	2.6±0.2	7.2	4.77	0.014
		Cu-O	1.85±0.02	1.6±0.2	7.2		
7Cu-Stable	8979.2	Cu-Cu	2.54±0.02	4.8±0.5	9.7	4.53	0.007
		Cu-O	1.85±0.02	1.3±0.1	5.2		
9Cu-Stable	8979.0	Cu-Cu	2.53±0.02	8.2±0.8	9.3	3.10	0.009
		Cu-O	1.82±0.02	0.4±0.1	7.8		
11Cu-Stable	8979.0	Cu-Cu	2.53	10.5	9.5	3.47	0.002
		Cu-O	-	-	-		
Cu[f]	8979.0	Cu-Cu	2.56	12			
		Cu-O					
Cu_2O[f]	8980.8	Cu-Cu	3.70	8			
		Cu-O	1.85	4			
CuO[f]	8983.8	Cu-Cu	2.91	4			
		Cu-O	1.91	2			
		Cu-O	1.99	2			

[a]R is interatomic distance. [b]CN is coordination number. [c]σ^2 is Debye-Waller factor, a measure of thermal and static disorder in absorber scatter distances. [d]ΔE_0 is edge energy shift. [e]No reliable fitted values can be obtained. [f]EXAFS data (R and CN) were calculated by FEFF code from the reported crystal structures[32] (JCPDS card#: 4-836, 5-667 and 41-254 for Cu, Cu_2O, and CuO, respectively)

Supplementary Table 4. Catalytic performance of reported Cu-based catalysts for DMO hydrogenation to MG.

Catalysts	Temperature (°C)	H_2/DMO	Pressure (MPa)	DMO conversion%	MG selectivity%	Ref.
Cu/SiO_2-SP	240	150	3	15	85	6
	290	150	3	100	40	
Cu/AC	220	120	2.5	83	92	7
	260	120	2.5	95	55	
Cu/HAP	190	150	2.5	3	95	8
	220	150	2.5	95	50	
Cu/MOF	170	50	2	9	93	9
	190	50	2	98	50	
Cu/NAHS	195	70	2.5	90	54	10
Cu/ZrSi	190	150	3	100	1	11
Cu/MMO	165	50	2	100	3	12
Cu/Cordierite	200	80	2.5	95	5	13
Cu/SiO_2-AE	200	80	2.5	100	4	14
5Cu-Stable	200	80	2.5	100	95	This work

Supplementary References

1. Yue, H., Zhao, Y., Ma, X. & Gong, J. Ethylene glycol: Properties, synthesis, and applications. *Chem. Soc. Rev.* **41**, 4218-4244 (2012).
2. Gong, J. et al. Synthesis of ethanol via syngas on Cu/SiO_2 catalysts with balanced Cu^0-Cu^+ sites. *J. Am. Chem. Soc.* **134**, 13922-13925 (2012).
3. Xu, C. et al. Interfacing with silica boosts the catalysis of copper. *Nat. Commun.* **9**, 3367 (2018).
4. Witzke, M., Dietrich, P., Ibrahim, M., Al-Bardan, K., Triezenberg, M. & Flaherty, D. Spectroscopic evidence for origins of size and support effects on selectivity of Cu nanoparticle dehydrogenation catalysts. *Chem. Commun.* **53**, 597-600 (2017).
5. Wang, Y., Shen, Y., Zhao, Y., Lv, J., Wang, S. & Ma, X. Insight into the balancing effect of active Cu species for hydrogenation of carbon-oxygen bonds. *ACS Catal.* **5**, 6200-6208 (2015).
6. Sun, J. et al. Freezing copper as a noble metal–like catalyst for preliminary hydrogenation. *Sci. Adv.* **4**, eaau3275 (2018).
7. Cui, Y., Wang, B., Wen, C., Chen, X. & Dai, W. Investigation of activated-carbon-supported copper catalysts with unique catalytic performance in the hydrogenation of dimethyl oxalate to methyl glycolate. *ChemCatChem* **8**, 527-531 (2016).
8. Wen, C., Cui, Y., Chen, X., Zong, B. & Dai, W. Reaction temperature controlled selective hydrogenation of dimethyl oxalate to methyl glycolate and ethylene

glycol over copper-hydroxyapatite catalysts. *Appl. Catal. B-Environ.* **162**, 483-493 (2015).

9. Ye, R. et al. Synthesis of robust MOF-derived Cu/SiO_2 catalyst with low copper loading via sol–gel method for the dimethyl oxalate hydrogenation reaction. *ACS Catal.* **8**, 3382-3394 (2018).

10. Yao, D., Wang, Y., Li, Y., Zhao, Y., Lv, J. & Ma, X. A high-performance nanoreactor for carbon–oxygen bond hydrogenation reactions achieved by the morphology of nanotube-assembled hollow spheres. *ACS Catal.* **8**, 1218-1226 (2018).

11. Zhu, Y., Kong, X., Cao, D., Cui, J., Zhu, Y.& Li, Y. The rise of calcination temperature enhances the performance of Cu catalysts: Contributions of support. *ACS catal.* **4,** 3675-3681 (2014)

12. Cui, G. et al. Low-temperature hydrogenation of dimethyl oxalate to ethylene glycol via ternary synergistic catalysis of Cu and acid-base sites. *Appl. Catal. B: Environ.* **248**, 394-404 (2019).

13. Yue, H. et al. Hydrogenation of dimethyl oxalate to ethylene glycol on a Cu/SiO_2/cordierite monolithic catalyst: enhanced internal mass transfer and stability. *AIChE J.* **58**, 2798-2809 (2012).

14. Gong, J. et al. Synthesis of ethanol via syngas on Cu/SiO_2 catalysts with balanced Cu^0-Cu^+ sites. *J. Am. Chem. Soc.* **134**, 13922-13925 (2012).
